# Deformylation reaction-based probe for *in vivo* imaging of HOCl†
†Electronic supplementary information (ESI) available: Movie S1 (naked-eye detection in aqueous solution); Movie S2 (*in vivo* NIR fluorescence imaging) and Movie S3 (*Z*-scan images of frozen sections isolated from the arthritic area). Crystal data for **FDOCl-1** (CCDC number: 1555875; CIF); crystal data for **FDOCl-4** (CCDC number: 1555871; CIF). CCDC 1555875 and 1555871. For ESI and crystallographic data in CIF or other electronic format see DOI: 10.1039/c7sc03784h


**DOI:** 10.1039/c7sc03784h

**Published:** 2017-11-03

**Authors:** Peng Wei, Wei Yuan, Fengfeng Xue, Wei Zhou, Ruohan Li, Datong Zhang, Tao Yi

**Affiliations:** a Department of Chemistry , Collaborative Innovation Center of Chemistry for Energy Materials , Fudan University , 220 Handan Road , Shanghai 200433 , China . Email: yitao@fudan.edu.cn; b Shandong Provincial Key Laboratory of Fine Chemicals , School of Chemistry and Pharmaceutical Engineering , Qilu University of Technology , Jinan 250353 , Shandong , China

## Abstract

The detection of hypochlorous acid (HOCl) *in vivo* is vitally important because the local concentration of HOCl is highly correlated with some diseases such as atherosclerosis and rheumatoid arthritis. However, *in vivo* detection of HOCl remains a challenge due to the lack of a suitable probe. We report here a near-infrared (NIR) emissive “turn-on” probe (**FDOCl-1**) based on a methylene blue derivative, which can quickly detect HOCl *via* a newly found deformylation mechanism. **FDOCl-1** displays remarkable selectivity and sensitivity towards HOCl. The dramatic changes in colour and NIR emission were used to detect HOCl *in vitro* and *in vivo* in a mouse arthritis model.

## Abstract 2

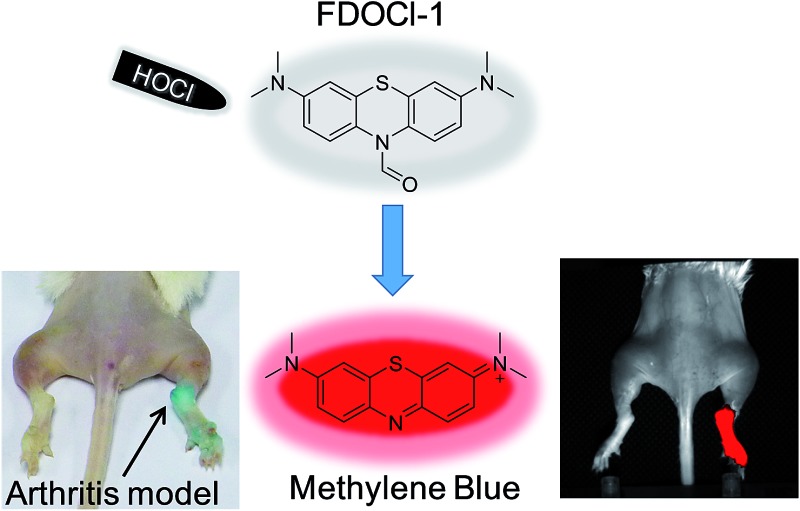
We report a near-infrared emissive probe which can detect HOCl *in vivo* by both fluorescence imaging and the naked eye.

## Introduction

Reactive oxygen species (ROS) are attracting increasing attention because of their essential role in many biological processes including signal transduction, inflammation and carcinogenesis and in neurodegenerative diseases.^1^–^3^ For many years we have focused on developing novel ROS-identifying fluorescent probes and have used these to successfully detect biomarkers (*e.g.* H_2_O_2_ and ˙OH) in biological systems.^4^–^7^ Hypochlorous acid (HOCl), an ROS produced by activated neutrophils, is an extremely important bactericidal oxidant *in vivo* and plays a protective role in human health.^8^ Excessive amounts of HOCl can, however, react rapidly with amine-containing side chains in some proteins, thereby altering the function of the protein and potentially contributing to the development of diseases such as atherosclerosis and rheumatoid arthritis.^9^–^11^ Methods for directly visualizing HOCl and rapidly quantifying HOCl levels *in vivo* are, therefore, of vital importance for disease diagnosis.

**Scheme 1:**
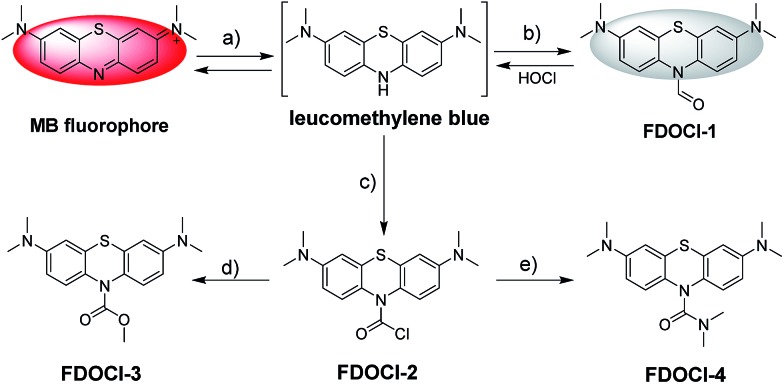
Structures and detecting mechanisms of prepared probes for HOCl. The reagents are: (a) Na_2_S_2_O_4_ and Na_2_CO_3_; (b) the Vilsmeier–Haack reagent; (c) bis(trichloromethyl) carbonate; (d) 4-dimethylaminopyridine, Na_2_CO_3_ and MeOH; and (e) Na_2_CO_3_ and dimethylamine.

Many fluorescent probes have been developed to detect HOCl *ex vivo*. These probes use different mechanisms including oxidation of substituted phenol analogues,^12^–^15^ deoximation of luminescent oximes,^16^–^19^ oxidation of thioethers to sulfonates or selenides to selenoxides,^20^–^28^ chlorination of thioesters or amides^29^–^33^ and cleavage of carbon–carbon double bonds (Table S1†).^34^,^35^ Although most probes have been successfully used to image low concentrations of intracellular HOCl, few probes have been used to detect HOCl in living organisms (Table S1†). Moreover, none of the reported probes are able to identify HOCl *in vivo* by both fluorescence imaging and the naked eye because of their low sensitivity and molar absorptivity in the visible range. Indeed, it is rare for colorimetric probes to provide significant absorption changes at biocompatible concentrations (Table S1 in the ESI†). These considerations encouraged us to develop a new probe (**FDOCl-1** in Scheme 1) for *in vivo* sensing of HOCl with optimal fluorescence and absorption changes, based on a newly found deformylation reaction. To our knowledge, this is the first example of using a deformylation reaction strategy to design probes for the detection of HOCl.

## Experimental section

### Materials

All starting materials were obtained from commercial suppliers and used as received. Methylene blue, sodium hydrosulfite and methyl iodide were purchased from J&K Chemical Technology. 4-Dimethylaminopyridine was purchased from Energy Chemical Company. Other general chemicals were purchased from Adamas-beta; Beijing InnoChem Science & Technology Co., Ltd; Aldrich Chemical Co.; Dojindo; and Sinopharm Chemical Reagent Co., Ltd. All organic solvents were supplied by Strong Chemical Company (Shanghai, China). MTT and cell culture reagents were purchased from Invitrogen.

All experiments were performed in compliance with the relevant laws and institutional guidelines. All animal experiments were performed according to procedures approved by the Fudan University Committee on Animal Care and Use. Informed consent was obtained for any experimentation with human subjects.

### Instruments

^1^H NMR (400 MHz) and ^13^C NMR (100 MHz) were carried out on a Bruker AV400 nuclear magnetic resonance spectrometer using DMSO-d_6_, CDCl_3_ or CD_3_CN as the solvent. Proton chemical shifts are reported in parts per million downfield from tetramethylsilane (TMS), with tetramethylsilane (*δ* = 0.0 ppm) or the solvent residue peak for CDCl_3_ (7.26 ppm for ^1^H and 77.16 ppm for ^13^C), CD_3_CN (1.94 ppm for ^1^H and 1.32 ppm for ^13^C) or DMSO-d_6_ (2.50 ppm for ^1^H and 39.52 ppm for ^13^C) as the chemical shift standard. High-resolution mass spectra (HRMS) were obtained on a Bruker Micro TOF II 10257 instrument with the electro-spray ionization (ESI) technique and direct injection method. High-performance liquid chromatography (HPLC) was performed using a Thermo Scientific Dionex Ultimate 3000 system with a diode array detector. Single crystals suitable for X-ray diffraction study were obtained at room temperature. X-ray intensity data were collected at 173 K on a CCD-Bruker SMART APEX system. UV-visible spectra were recorded on a Shimadzu UV-2550 spectrometer. Steady-state fluorescence spectra at room temperature were obtained on an Edinburgh instrument FLS-920 spectrometer with a Xe lamp as an excitation source. Time-dependent studies and absolute fluorescence quantum yield (*Φ*) measurements were carried out using a PTI QM40. Electrochemical experiments were carried out in a conventional three-electrode system using an electrochemical analyzer (CHI 660E, CHI Shanghai, Inc). Ag/AgCl, platinum wire and glassy carbon electrodes were used as the reference electrode, counter electrode and working electrode, respectively.

### Preparation of probes and analytes

Stock solutions of the probes (1 mM) were prepared in ethanol. The test solutions of the probes (10 μM) in 10 mM PBS solution (pH = 7.2) were prepared by placing stock solutions in cuvettes and diluting with buffer solution to the test concentration. The resulting solutions were shaken well and incubated at room temperature before recording the spectra.

HOCl was obtained from 14.5% NaOCl solution. Other ROS/RNS (25–100 μM) were prepared in ddH_2_O. H_2_O_2_ was diluted from a 30% solution. TBHP (*tert*-butyl hydroperoxide) was obtained from a 70% TBHP solution in ddH_2_O. ROO˙ was prepared by dissolving 2,2′-azobis(2-amidinopropane)dihydrochloride in ddH_2_O. NO was prepared by dissolving SNP (sodium nitroferricyanide(iii)dihydrate) in ddH_2_O. O_2_^–^ was prepared by dissolving KO_2_ (potassium superoxide) in ddH_2_O. ˙OH (the hydroxyl radical) was generated by the Fenton reaction. To generate ˙OH, H_2_O_2_ was added in the presence of 10 equiv. of ferrous chloride. The concentration of ˙OH was equal to the H_2_O_2_ concentration. ONOO^–^ was prepared using 3-morpholinosydnonimine hydrochloride. *t*-BuOO˙ was prepared by adding TBHP in the presence of 10 equiv. of ferric perchlorate hydrate. The concentration of *t*-BuOO˙ was equal to the TBHP concentration. Other analytes were prepared in ddH_2_O. Unless otherwise noted, for all fluorescence measurements, the excitation wavelength was 620 nm and the emission wavelength was collected from 640 to 810 nm.

### Detection limit

The detection limit (3*σ*/*k*) was calculated based on the linear relationship between the fluorescence intensity at 686 nm or absorbance at 664 nm and the concentration of HOCl. *σ* is the standard deviation of the blank measurement (*n* = 11) and *k* is the slope of the fluorescence intensity or absorbance *versus* HOCl concentration.

### Cell culture

RAW264.7 macrophages were provided by the Institute of Biochemistry and Cell Biology, SIBS. The cells were cultured in phenol red-free Dulbecco’s modified essential medium (RPMI 1640) supplemented with 10% fetal bovine serum (FBS) and 1% Pen–Strep. The cells were incubated at 37 °C under 5% CO_2_ and split with trypsin/EDTA solution (0.25%) as recommended by the manufacturer.

### MTT assay

The methyl thiazolyl tetrazolium (MTT) assay was used to detect the cytotoxicity of **FDOCl-1**. Cells were seeded in 96-well plates at a density of 1 × 10^4^ cells per well and then cultured in 5% CO_2_ at 37 °C for 24 h. After the cells were incubated with **FDOCl-1** at different concentrations (0, 5, 10, 15, 20, 25, 30, 35 and 40 μM in DMSO/cell culture medium with 10% FBS = 1 : 49) for 6 and 12 h, MTT (20 μL, 5 mg mL^–1^) was added to each well of the 96-well assay plate for 4 h at 37 °C. After dimethyl sulfoxide (DMSO, 100 μL per well) was added, the absorbance was measured at 490 nm using a microplate reader. All samples were analyzed in triplicate.

### CLSM imaging

RAW 264.7 macrophages (5 × 10^8^ per mL) were plated on 14 mm glass coverslips and allowed to adhere for 24 h. The cells were then incubated with different analytes for a pre-set time at 37 °C. After incubation, the cells were washed three times with PBS. Frozen sections were prepared using a Cryostar NX50 Cryostat according to the reported procedure.^36^ CLSM imaging was performed on an Olympus FV1000 confocal scanning system with a 60× and 20× oil-immersion objective lens for cells and frozen sections, respectively. Red channel: 700 ± 50 nm, *λ*_ex_ = 633 nm.

### *In vivo* imaging of HOCl in a mouse model

The animal procedures were in agreement with the guidelines of the Institutional Animal Care and Use Committee. Arthritis was generated by injecting different volumes of λ-carrageenan (5 mg mL^–1^, in PBS) into the right tibiotarsal joints (right ankles) of 8–10 week-old mice. No λ-carrageenan was injected into the left tibiotarsal joints (left ankles) in order to generate a control group. After four hours, the left and right ankles were injected with the same amount of **FDOCl-1** (100 μL, 1 mM). In the small animal *in vivo* fluorescence imaging system, an adjustable 0.3 mW 635 nm continuous wavelength laser (Connet Fiber Optics, China) was used as the excitation source, and the fluorescence signal was collected using an Andor DU897 EMCCD with a Semrock 720 ± 60 nm bandpass filter.

## Results and discussion

### Design of the probe

Selecting a suitable fluorophore is the first important step in designing an ideal probe that can identify HOCl *in vivo*. Methylene blue (MB) is a Food and Drug Administration (FDA) approved drug for indications such as malaria, methemoglobinemia and cyanide poisoning in humans, and is often used as a tissue staining dye for visible imaging.^37^–^41^ MB is a near-infrared (NIR) fluorophore (*λ*_em_ > 600 nm) and has strong absorption in aqueous solution at wavelengths of 550–700 nm (maximum at 664 nm, *ε* = 85 000 M^–1^ cm^–1^).^40^,^41^ The reduced form of MB (leucomethylene blue, LMB), however, is non-fluorescent and absorbs only in the ultraviolet region.^42^ Oxidation of LMB or its derivatives generates intense absorption changes, with concomitant NIR emission. LMB and its derivatives are thus ideal scaffolds for the construction of probes that can identify specific analytes using both fluorescence and absorption changes. Only two previous reports describe the use of LMB derivatives as fluorescent sensors (of nitroreductase and palladium).^43^,^44^ The *in vivo* application of such probes has not, however, been reported.

**Fig. 1:**
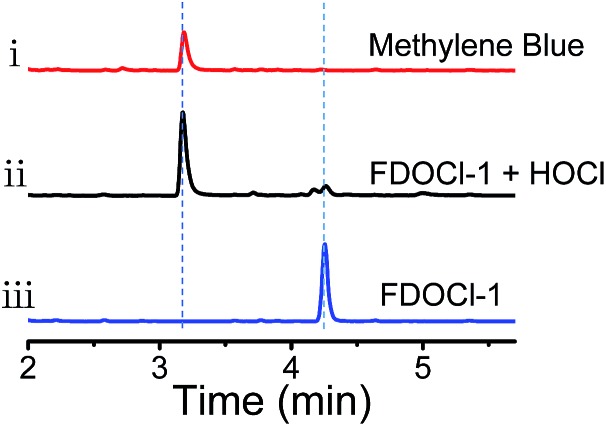
HPLC analysis of the aqueous solution from (i) 10 μM methylene blue, (ii) 10 μM **FDOCl-1** + 25 μM HOCl and (iii) 10 μM **FDOCl-1** (254 nm).

Herein, four different LMB derivatives were designed and synthesized (**FDOCl-1–FDOCl-4** in Scheme 1, synthetic details are shown in the ESI†). The formyl derivative of LMB, **FDOCl-1** (crystal structure shown in Fig. S1† and crystal data and structure refinement details shown in Table S2†), was rapidly (<30 s) deformylated by HOCl under mild conditions to regenerate MB, with marked changes in colour and NIR emission. Both HPLC and HRMS analysis confirmed the formation of MB (Fig. 1 and S2†). **FDOCl-2** (the carbamoyl chloride derivative) and **FDOCl-3** (the methyl carbamate derivative) were found to be very stable in the presence of HOCl (Scheme 1 and Fig. S3†). Although **FDOCl-4** (the dimethylamino carbamate derivative, crystal structure shown in Fig. S1† and crystal data and structure refinement details shown in Table S2†) did react with HOCl, the reaction required a much longer time (>10 min) (Fig. S4†).

### The reaction mechanism of the probes towards HOCl

**Scheme 2:**
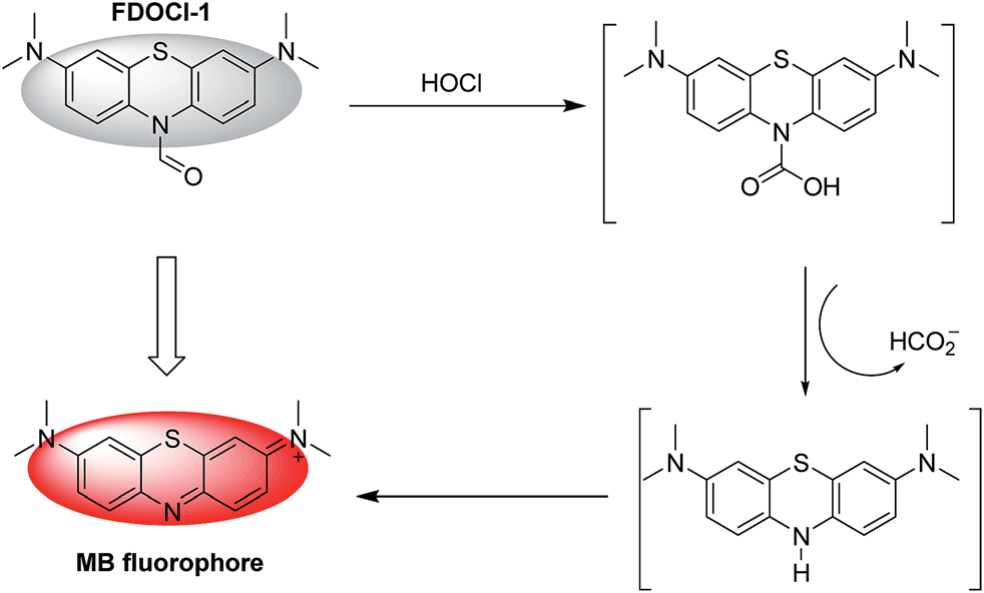
The proposed mechanism of the reaction of **FDOCl-1** with HOCl.

The proposed mechanism by which **FDOCl-1** detects HOCl is shown in Scheme 2. In the first step of the reaction, the aldehyde group of **FDOCl-1** was oxidized to carboxylic acid by HOCl to form a relatively unstable carbamic acid derivative. This would then hydrolyze quickly in aqueous solution to form the unstable LMB, which would be oxidized to MB. The difference in reactivity among the four compounds is due to their different redox potentials, which were confirmed by electrochemical studies (cyclic voltammetry) in CH_2_Cl_2_ containing 0.1 M tetrabutylammonium hexafluorophosphate (TBAPF6) (Fig. S5†). The electrochemical data revealed that (1) among the four compounds, the sequence of reductive reactivity is **FDOCl-1** > **FDOCl-4** ≫ **FDOCl-3** > **FDOCl-2**, which is consistent with the reactivity towards HOCl and (2) a weak reductive peak at –0.161 V was observed for **FDOCl-1** but no oxidative peak was detected in the reverse direction, indicating that the oxidation of **FDOCl-1** was not reversible. This result was consistent with the reaction mechanism shown in Scheme 2 in which **FDOCl-1** is first oxidized and then quickly decarboxylated in the reaction. These data indicate that the selective deformylation of **FDOCl-1** by HOCl could be used as a novel strategy for detecting HOCl.

### The high sensitivity and selectivity of **FDOCl-1** for HOCl

**Fig. 2:**
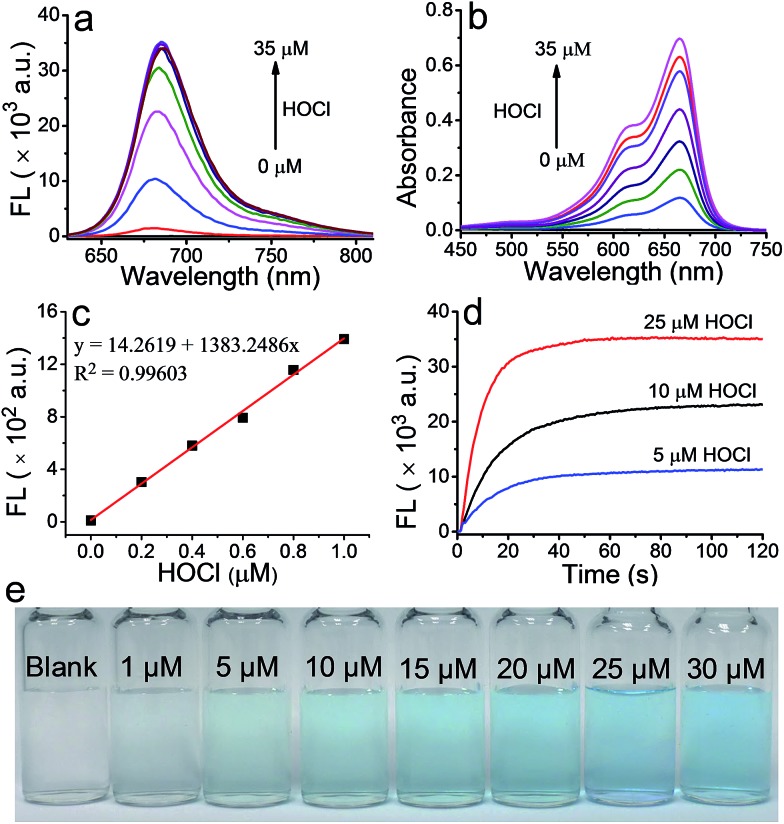
(a) Fluorescence and (b) absorption spectra of **FDOCl-1** (10 μM in 10 mM PBS, pH 7.2) in the presence of different concentrations of HOCl; (c) the linear relationship between the fluorescence intensity at 686 nm and the concentration of HOCl; (d) time-dependent changes in the fluorescence intensity of **FDOCl-1** (10 μM) at 686 nm after adding different concentrations of HOCl; and (e) colour changes of **FDOCl-1** (10 μM) after adding different concentrations of HOCl (time range 0–120 s, *λ*_ex_ = 620 nm).

The ability of **FDOCl-1** to detect HOCl was evaluated by spectroscopy under simulated physiological conditions (10 mM sodium phosphate buffer (PBS), pH 7.2 and 0.1% EtOH). As expected, neither fluorescence nor absorption by **FDOCl-1** was detected in the visible region since the electronic communication between the two aniline moieties was interrupted, thus breaking the conjugation system of the compound. After treatment with HOCl (25 μM, 2.5 equiv.), the fluorescence intensity of **FDOCl-1** at 686 nm increased 2068-fold (Fig. 2 and S6†) and the absorbance at 664 nm increased 577-fold (Table S3 and S6†). The fluorescence quantum yield and brightness of **FDOCl-1** after reaction with HOCl are 2.0% and 1154 M^–1^ cm^–1^, respectively (Table S7†).^45^ The variations in fluorescence and absorbance of **FDOCl-1** in the presence of HOCl may be the largest among the reported probes because of the rapid deformylation of **FDOCl-1**. Notably, even low levels (1 μM, 0.1 equiv.) of HOCl induced a 78-fold increase in the fluorescence intensity of **FDOCl-1** (Table S3†). The detection limits for HOCl, evaluated by the changes in absorption and fluorescence of **FDOCl-1**, were as low as 3.98 nM and 2.62 nM, respectively (Fig. 2c and S7†), which are lower than those of most of the HOCl sensors, but slightly higher than the best reported one (HKOCl-3 in Table S1†).^13^ These data illustrate the extreme sensitivity of **FDOCl-1** to HOCl. The reaction of **FDOCl-1** with HOCl was complete within 30 s (Fig. 2d, Movie S1†), under pseudo-first-order conditions, giving an observed rate constant of 0.1011 s^–1^ (Fig. S8†). With the addition of increasing amounts of HOCl, the solution of **FDOCl-1** gradually developed a blue colour that could be clearly observed by the naked eye (Fig. 2e).

**Fig. 3:**
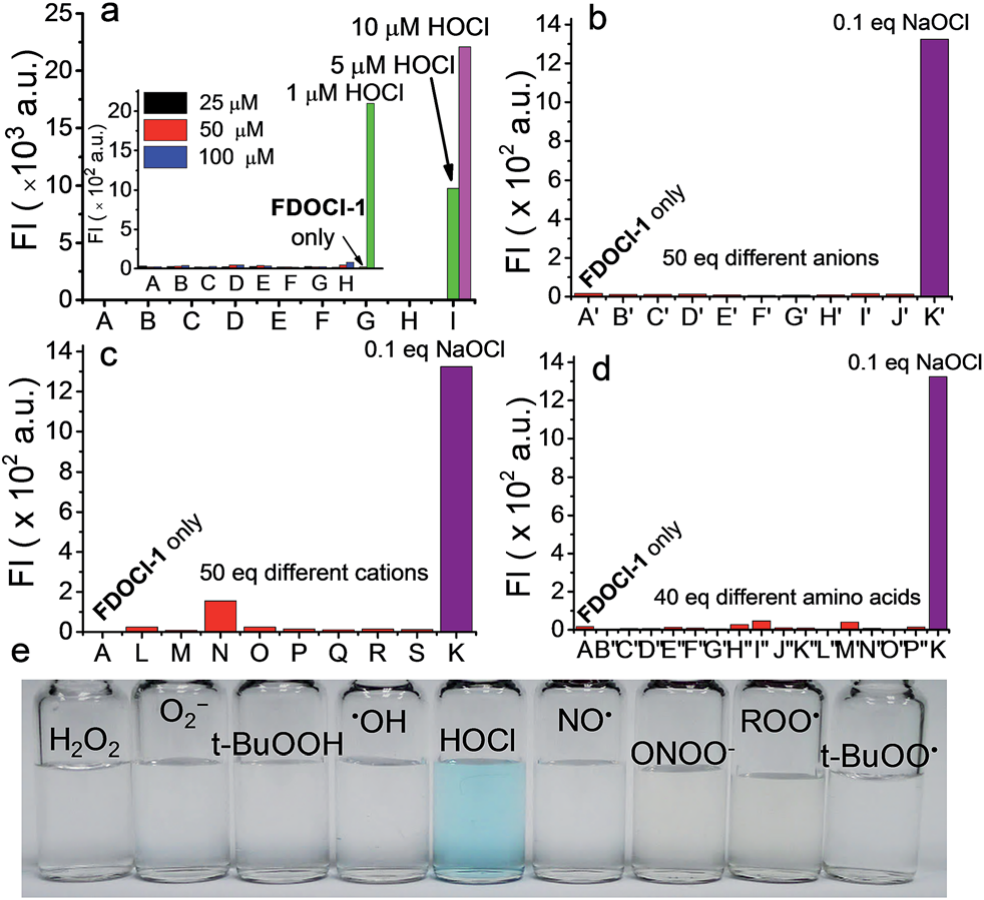
Fluorescence intensity of **FDOCl-1** (10 μM in 10 mM PBS, pH 7.2) at 686 nm after (a) adding various ROS/RNS (from (A) to (H): H_2_O_2_, O^2–^, *t*-BuOOH, ˙OH, NO, ONOO^–^, ROO˙ and *t*-BuOO˙ with concentrations of 25, 50 and 100 μM and (I): HOCl with a concentration of 1, 5 and 10 μM; the inset shows magnified data comparing A to H with 1 μM HOCl), (b) adding various anions (from (A′) to (K′): blank, CH_3_COO^–^, CO_3_^2–^, SO_4_^2–^, Cl^–^, ClO_4_^–^, F^–^, I^–^, NO_2_^–^, S_2_O_3_^2–^ and OCl^–^), (c) adding various cations (from (L) to (S): Al^3+^, Ca^2+^, Cu^2+^, Fe^3+^, K^+^, Mg^2+^, NH_4_^+^ and Ni^+^) and (d) adding various amino acids (from (B′′) to (P′′): Leu, Pro, Gly, Gln, Glu, Met, Lys, Trp, Ser, Thr, Asp, Ile, Val, His and Ala). (e) Colour changes of **FDOCl-1** (10 μM) after adding HOCl (25 μM) and other different ROS/RNS (100 μM) with *λ*_ex_ = 620 nm.

A high level of selectivity is of paramount importance for an effective chemosensor. To verify the selectivity of **FDOCl-1** for HOCl, both fluorescence and absorption changes were recorded upon addition of HOCl and other analytes. Changes in the fluorescence intensity of **FDOCl-1** (10 μM in 10 mM PBS, pH 7.2 and 0.1% EtOH) in the presence of 10 μM (1 equiv.) HOCl were >631-fold greater than in the presence of 100 μM (10 equiv.) of similar ROS/RNS, including H_2_O_2_, O_2_^–^, *t*-BuOOH, NO, ROO˙ and ONOO^–^ (Table S4†). This selectivity is higher than for previously reported HOCl probes (Table S1†). Even highly reactive oxygen radicals, such as ˙OH and *t*-BuOO˙, did not noticeably enhance the fluorescence intensity of **FDOCl-1** (Fig. 3a and Table S4†). The reactivity of **FDOCl-1** towards some common anions, cations and biological substances was also tested. Neither the addition of 50 equiv. of common anions and cations, such as CH_3_COO^–^, CO_3_^2–^, SO_4_^2–^, Cl^–^, ClO_4_^–^, F^–^, I^–^, NO_2_^–^, S_2_O_3_^2–^, Al^3+^, Ca^2+^, Cu^2+^, Fe^3+^, K^+^, Mg^2+^, NH_4_^+^ and Ni^+^, nor 40 equiv. of amino acids, such as Leu, Pro, Gly, Gln, Glu, Met, Lys, Trp, Ser, Thr, Asp, Ile, Val, His and Ala, caused a noticeable enhancement of the fluorescence intensity of **FDOCl-1** (Fig. 3b–d). The fact that none of these tested analytes caused a significant change in the absorption spectrum further confirmed the superior selectivity of **FDOCl-1** towards HOCl (Table S6 and Fig. S9 and S10†). Notably, only HOCl induced a blue colour change that could be clearly observed by the naked eye (Fig. 3e and S11–S13†).

To guarantee the application of **FDOCl-1** for the detection of HOCl *in vivo*, the interference of some cellular reductants, such as sulfhydryl compounds (glutathione (GSH) and *N*-acetylcysteine (NAC)), and aldehyde containing compounds (aldehyde and glucose) was studied.^46^,^47^ As shown in Fig. S14,† sulfhydryl compounds such as GSH and NAC may affect the response of **FDOCl-1** to HOCl because both of the compounds can react with HOCl and consume HOCl to some extent. However, even in the presence of 10 eq. of GSH or NAC (100 μM), 1.0 eq. HOCl could induce an obvious fluorescence intensity increase of **FDOCl-1** (8-fold in the case of GSH and 33-fold in the case of NAC compared to **FDOCl-1** itself). Meanwhile, high concentrations of aldehyde containing compounds such as aldehyde and glucose have very little impact on the reaction of HOCl towards **FDOCl-1**. These results suggest that **FDOCl-1** could be used to detect HOCl reliably in complex cellular milieu. Moreover, **FDOCl-1** was shown to be stable in the pH range of 4–9 and its selectivity was not influenced by pH in this range (Fig. S15 and S16†). The fluorescent product of **FDOCl-1** (MB) could remain stable in a common cell medium in the presence of a large excess of HOCl (10 μM MB in the presence of 20 equiv. HOCl) for one hour (Fig. S17†). Thus, **FDOCl-1** is suitable for detecting HOCl/NaOCl in a wide variety of biological environments.

### Evaluation of **FDOCl-1** for HOCl detection in live cells

**Fig. 4:**
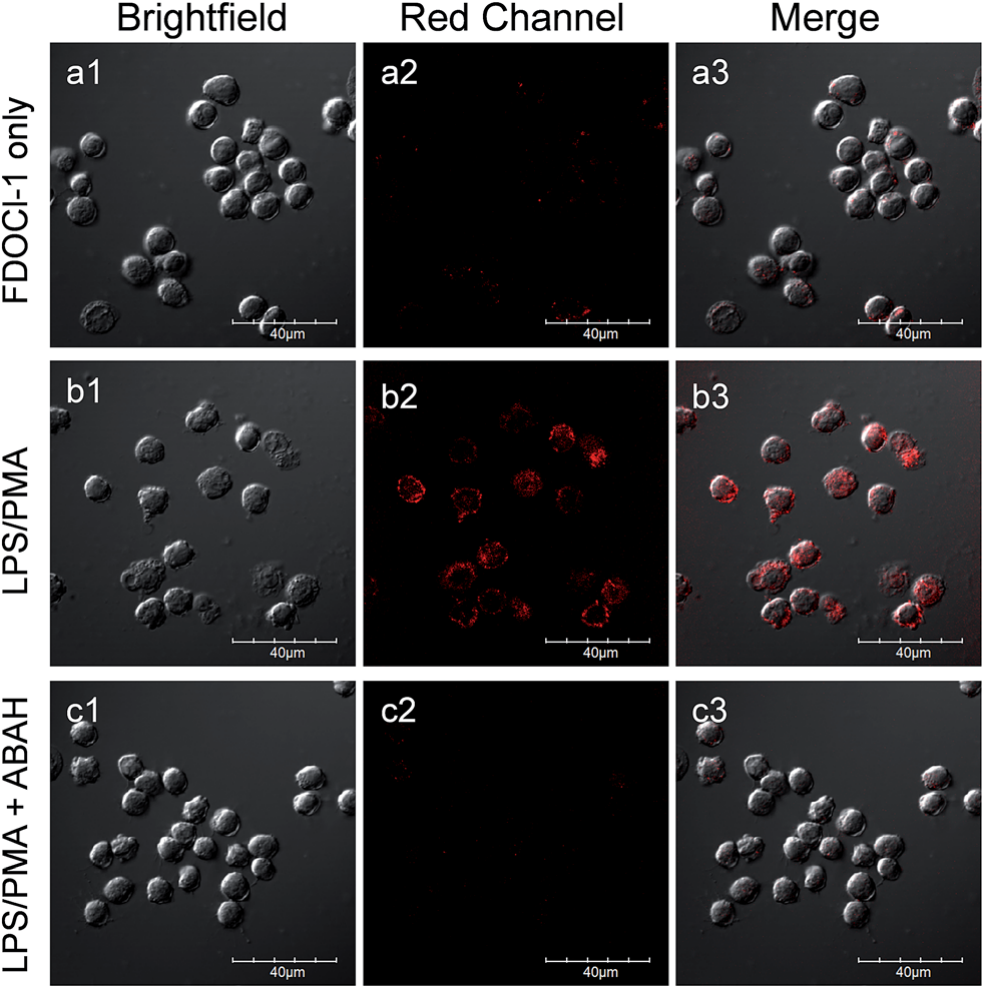
CLSM images of live RAW 264.7 macrophages incubated with **FDOCl-1** (10 μM) for 60 min, washed with PBS buffer (a1–a3) then stimulated with (b1–b3) LPS (1 μg mL^–1^)/PMA (500 ng mL^–1^) or (c1–c3) LPS (1 μg mL^–1^)/PMA (500 ng mL^–1^)/ABAH (250 μM) for 1 h. CLSM imaging was performed on an Olympus FV1000 confocal scanning system with a 60× immersion objective lens. Red channel: 700 ± 50 nm, *λ*_ex_ = 633 nm.

Because of its high signal to noise ratio, excellent selectively and rapid response time towards HOCl, **FDOCl-1** should be a suitable probe for *in vivo* detection of HOCl. To evaluate the compatibility of **FDOCl-1** with biological systems, we examined the cytotoxicity of **FDOCl-1** in RAW 264.7 macrophages using the methyl thiazolyl tetrazolium (MTT) assay. The viability of the macrophages was >99% after incubation with **FDOCl-1** (40 μM) for 12 h, indicating that **FDOCl-1** has minimal cytotoxicity (Fig. S18†). To assess the capability of **FDOCl-1** to detect HOCl in cells, RAW 264.7 macrophages loaded with **FDOCl-1** (10 μM) were treated with different concentrations of exogenous and endogenous HOCl, respectively. Cell images were then obtained using confocal laser scanning microscopy (CLSM). As shown in Fig. S19,† RAW 264.7 macrophages incubated with **FDOCl-1** showed no fluorescence. However, after treating with HOCl, the cells show a remarkable fluorescence intensity increase in the cytoplasm and the fluorescence intensity was dependent on the concentration of HOCl. Further study showed that **FDOCl-1** could also detect endogenous HOCl stimulated by lipopolysaccharides (LPS) and phorobol myristate acetate (PMA). In the experiment, RAW 264.7 macrophages were incubated with **FDOCl-1** then treated with LPS and PMA to induce endogenous HOCl. As shown in Fig. S20† and 4, the remarkable fluorescence increase with the increasing concentration of PMA and LPS reflected the generation of endogenous HOCl. 4-Aminobenzoic acid hydrazide (ABAH), a myeloperoxidase (MPO) inhibitor, which could decrease the HOCl level, was also added to generate control experiments.^48^,^49^ As shown in Fig. 4c, the fluorescence intensity of the stimulated cells was suppressed when the cells were coincubated with 250 μM ABAH.

The photostability of the fluorescent product MB was also evaluated as shown in Fig. S21.† The fluorescence intensity of MB decreased by about 25% after 10 min of exposure to the laser. This photostability was much better than that of the commercial NIR emissive dye Cy5 whose fluorescence intensity decreased by about 78% when exposed to a laser under the same conditions. Meanwhile, MB could remain in cells for more than 1 hour (Fig. S23†). All these data show that **FDOCl-1** is cell permeable and can be used to detect HOCl in living cells.

### *In vivo* imaging of arthritis-dependent HOCl production

**Fig. 5:**
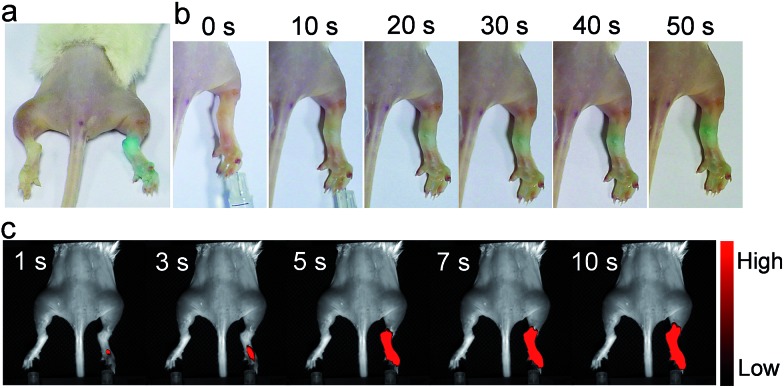
*In vivo* images of the mouse model of arthritis. Colour changes observed by the naked eye (a) more than 2 min after injection of **FDOCl-1** and (b) 0–50 s after injection of **FDOCl-1**; (c) fluorescence images taken 1–10 s after injection of **FDOCl-1**. The arthritis model was generated by injecting λ-carrageenan (100 μL, 5 mg mL^–1^ in PBS) into the right tibiotarsal joint (right ankle); the left tibiotarsal joint (left ankle) was used as a control. The fluorescence signal was collected at *λ*_em_ = 720 ± 60 nm under excitation by a 635 nm continuous wave (CW) laser with a power density of 0.3 mW cm^–2^; **FDOCl-1**: 100 μL and 1 mM.

With these *ex vivo* data in hand, we then used **FDOCl-1** for *in vivo* imaging in a λ-carrageenan-induced mouse model of arthritis. This model was chosen because HOCl plays an important role in joint destruction in rheumatoid arthritis.^9^ The arthritis was generated by injecting different volumes of λ-carrageenan (5 mg mL^–1^, in PBS) into the right tibiotarsal joints (right ankles) of 8–10 week-old mice. No λ-carrageenan was injected into the left tibiotarsal joints (left ankles) in order to generate the control group. After four hours the left and right ankles were injected with the same amount of **FDOCl-1** (100 μL, 1 mM) and only the arthritic paw area became blue within 30 s (Fig. 5a and b). In the control paws, without λ-carrageenan-induced arthritis, no colour change was observed, even 120 s after the injection of **FDOCl-1**. These data indicate that **FDOCl-1** can be used to identify HOCl in the arthritic area by the naked eye.

**Fig. 6:**
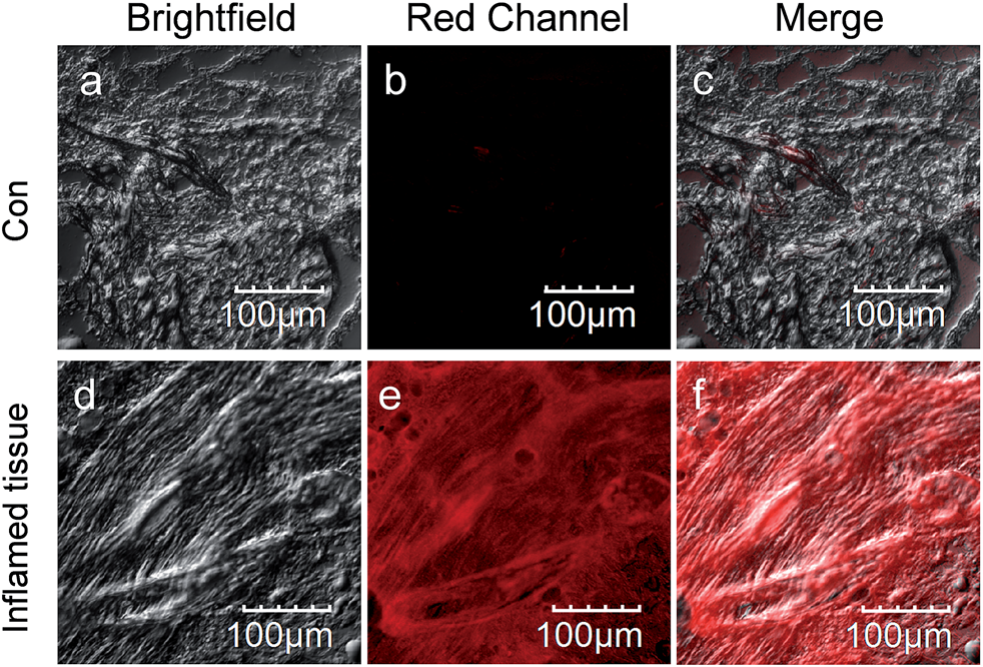
CLSM images of frozen sections prepared from mice with λ-carrageenan (100 μL, 5 mg mL^–1^ in PBS) induced arthritis. Inflamed tissue: sections isolated from the arthritic area. Con: sections isolated from the normal area. CLSM imaging was performed on an Olympus FV1000 confocal scanning system with a 20× immersion objective lens. Red channel: 700 ± 50 nm, *λ*_ex_ = 633 nm.

The response of **FDOCl-1** to HOCl *in vivo* was confirmed by fluorescence imaging (Fig. 5c and Movie S2†). The arthritic area of the mouse quickly showed strong fluorescence in the NIR range within 5 s (720 ± 60 nm), in contrast to the control side. Using **FDOCl-1** it was possible to correlate different levels of inflammation generated by different concentrations of λ-carrageenan with the intensity of the NIR emissions (Fig. S24†). In confocal laser scanning microscope images of frozen sections prepared from mice with λ-carrageenan-induced arthritis, sections isolated from the arthritic area showed strong fluorescence whereas those isolated from the controls showed almost no background interference (Movie S3† and Fig. 6). These findings indicate, for the first time, that **FDOCl-1** can detect arthritis-dependent HOCl production *in vivo*, by both fluorescence imaging and the naked eye.

## Conclusions

In conclusion, we have developed a new type of deformylation-based fluorescent probe, **FDOCl-1**, for the rapid detection of HOCl using both NIR emission and the naked eye *in vitro*. **FDOCl-1** exhibits high sensitivity and selectivity for HOCl at ultralow concentrations (UV: 3.98 nM; FL: 2.62 nM), guaranteeing its application for detecting HOCl/NaOCl in a wide variety of biological environments. The probe can be used to image the endogenous HOCl level generated in live RAW 264.7 macrophages through a cellular inflammation response. Additionally, the presence of HOCl *in vivo* can be easily identified by the naked eye using **FDOCl-1** without any signal amplifiers and the *in vivo* HOCl level can be estimated *via in vivo* images using NIR emission. Efforts are ongoing to develop clinical applications of **FDOCl-1** and to use this new probe to elucidate the production and transport of HOCl.

## Conflicts of interest

There are no conflicts to declare.

## Supplementary Material

Supplementary movie

Supplementary movie

Supplementary movie

Supplementary information

Crystal structure data

## References

[cit1] Chen X., Wang F., Hyun J. Y., Wei T., Qiang J., Ren X., Shin I., Yoon J. (2016). Chem. Soc. Rev..

[cit2] Nathan C. (2003). J. Clin. Invest..

[cit3] Yang Y., Zhao Q., Feng W., Li F. (2013). Chem. Rev..

[cit4] Meng L., Wu Y., Yi T. (2014). Chem. Commun..

[cit5] Wen Y., Liu K., Yang H., Li Y., Lan H., Liu Y., Zhang X., Yi T. (2014). Anal. Chem..

[cit6] Wen Y., Liu K., Yang H., Liu Y., Chen L., Liu Z., Huang C., Yi T. (2015). Anal. Chem..

[cit7] Wen Y., Xue F., Lan H., Li Z., Xiao S., Yi T. (2017). Biosens. Bioelectron..

[cit8] Hampton M. B., Kettle A. J., Winterbourn C. C. (1998). Blood.

[cit9] Hawkins C. L., Davies M. J. (2005). Chem. Res. Toxicol..

[cit10] Hawkins C. L., Pattison D. I., Davies M. J. (2003). Amino Acids.

[cit11] Summers F. A., Forsman Quigley A., Hawkins C. L. (2012). Biochem. Biophys. Res. Commun..

[cit12] Hu J. J., Wong N.-K., Gu Q., Bai X., Ye S., Yang D. (2014). Org. Lett..

[cit13] Hu J. J., Wong N.-K., Lu M.-Y., Chen X., Ye S., Zhao A. Q., Gao P., Yi-Tsun Kao R., Shen J., Yang D. (2016). Chem. Sci..

[cit14] Sun Z.-N., Liu F.-Q., Chen Y., Tam P. K. H., Yang D. (2008). Org. Lett..

[cit15] Zhou Y., Li J.-Y., Chu K.-H., Liu K., Yao C., Li J.-Y. (2012). Chem. Commun..

[cit16] Cheng X., Jia H., Long T., Feng J., Qin J., Li Z. (2011). Chem. Commun..

[cit17] Li D., Feng Y., Lin J., Chen M., Wang S., Wang X., Sheng H., Shao Z., Zhu M., Meng X. (2016). Sens. Actuators, B.

[cit18] Lin W., Long L., Chen B., Tan W. (2009). Chem.–Eur. J..

[cit19] Wu G., Zeng F., Wu S. (2013). Anal. Methods.

[cit20] Cheng G., Fan J., Sun W., Cao J., Hu C., Peng X. (2014). Chem. Commun..

[cit21] Kenmoku S., Urano Y., Kojima H., Nagano T. (2007). J. Am. Chem. Soc..

[cit22] Koide Y., Urano Y., Hanaoka K., Terai T., Nagano T. (2011). J. Am. Chem. Soc..

[cit23] Li G., Zhu D., Liu Q., Xue L., Jiang H. (2013). Org. Lett..

[cit24] Lou Z., Li P., Pan Q., Han K. (2013). Chem. Commun..

[cit25] Wu L., Wu I. C., DuFort C. C., Carlson M. A., Wu X., Chen L., Kuo C.-T., Qin Y., Yu J., Hingorani S. R., Chiu D. T. (2017). J. Am. Chem. Soc..

[cit26] Xu Q., Heo C. H., Kim G., Lee H. W., Kim H. M., Yoon J. (2015). Angew. Chem., Int. Ed..

[cit27] Yuan L., Wang L., Agrawalla B. K., Park S.-J., Zhu H., Sivaraman B., Peng J., Xu Q.-H., Chang Y.-T. (2015). J. Am. Chem. Soc..

[cit28] Zhang W., Liu W., Li P., kang J., Wang J., Wang H., Tang B. (2015). Chem. Commun..

[cit29] Cao L., Zhang R., Zhang W., Du Z., Liu C., Ye Z., Song B., Yuan J. (2015). Biomaterials.

[cit30] Chen X., Lee K.-A., Ha E.-M., Lee K. M., Seo Y. Y., Choi H. K., Kim H. N., Kim M. J., Cho C.-S., Lee S. Y., Lee W.-J., Yoon J. (2011). Chem. Commun..

[cit31] Chen X., Lee K.-A., Ren X., Ryu J.-C., Kim G., Ryu J.-H., Lee W.-J., Yoon J. (2016). Nat. Protoc..

[cit32] Xu Q., Lee K.-A., Lee S., Lee K. M., Lee W.-J., Yoon J. (2013). J. Am. Chem. Soc..

[cit33] Yuan L., Lin W., Xie Y., Chen B., Song J. (2012). Chem.–Eur. J..

[cit34] Chen S., Lu J., Sun C., Ma H. (2010). Analyst.

[cit35] Zou X., Liu Y., Zhu X., Chen M., Yao L., Feng W., Li F. (2015). Nanoscale.

[cit36] Bratthauer G. L. (2010). Methods Mol. Biol..

[cit37] Jiang Z., Duong T. (2016). Brain Circulation.

[cit38] Ormeci N., Savas B., Coban S., Palabıyıkoğlu M., Ensari A., Kuzu I., Kursun N. (2008). Surg. Endosc..

[cit39] Schirmer R. H., Adler H., Pickhardt M., Mandelkow E. (2011). Neurobiol. Aging.

[cit40] Junqueira H. C., Severino D., Dias L. G., Gugliotti M. S., Baptista M. S. (2002). Phys. Chem. Chem. Phys..

[cit41] Tardivo J. P., Del Giglio A., de Oliveira C. S., Gabrielli D. S., Junqueira H. C., Tada D. B., Severino D., de Fátima Turchiello R., Baptista M. S. (2005). Photodiagn. Photodyn. Ther..

[cit42] Wainwright M., Phoenix D. A., Rice L., Burrow S. M., Waring J. (1997). J. Photochem. Photobiol., B.

[cit43] Bae J., McNamara L. E., Nael M. A., Mahdi F., Doerksen R. J., Bidwell G. L., Hammer N. I., Jo S. (2015). Chem. Commun..

[cit44] Tan H.-y., Liu J.-g., Zhou L.-f., Li Y.-k., Yan J.-w., Zhang L. (2017). RSC Adv..

[cit45] Zhang Y., Swaminathan S., Tang S., Garcia-Amorós J., Boulina M., Captain B., Baker J. D., Raymo F. M. (2015). J. Am. Chem. Soc..

[cit46] Bai X., Huang Y., Lu M., Yang D. (2017). Angew. Chem..

[cit47] Hu J. J., Wong N.-K., Ye S., Chen X., Lu M.-Y., Zhao A. Q., Guo Y., Ma A. C.-H., Leung A. Y.-H., Shen J., Yang D. (2015). J. Am. Chem. Soc..

[cit48] Kettle A. J., Gedye C. A., Hampton M. B., Winterbourn C. C. (1995). Biochem. J..

[cit49] Kettle A. J., Gedye C. A., Winterbourn C. C. (1997). Biochem. J..

