# Supplementary material for: Deformylation reaction-based probe for in vivo imaging of HOCl
Source: Chem Sci. 2017 Nov 3;9(2):495–501. doi: 10.1039/c7sc03784h (PMC5868080; doi:10.1039/c7sc03784h)
Supplement: Supplementary file 4 [file SC-009-C7SC03784H-s004.pdf]

Electronic Supplementary Material (ESI) for Chemical Science.
This journal is © The Royal Society of Chemistry 2017

Electronic Supplementary Information for

Deformylation Reaction-Based Probe for *in vivo* Imaging of HOCl

Peng Wei,[†] Wei Yuan,[†] Fengfeng Xue,[†] Wei Zhou,[†] Ruohan Li,[†] Datong Zhang,[‡] and Tao Yi[*,†]

[†]Department of Chemistry and Collaborative Innovation Center of Chemistry for Energy Materials, Fudan University, 220 Handan Road, Shanghai 200433, China

[‡]Shandong Provincial Key Laboratory of Fine Chemicals, School of Chemistry and Pharmaceutical Engineering, Qilu University of Technology, Jinan 250353, Shandong, China

Content

1 Experiment Section 2
1.3 The synthesis of **FDOCl-1** 2
1.4 The synthesis of **FDOCl-2** 3
1.5 The synthesis of **FDOCl-3** 4
1.6 The synthesis of **FDOCl-4** 5
2 Additional tables 7
3 Additional images and figures 15
4 NMR and HRMS spectra 28
5 References 35

1 Experiment Section

1.1 The synthesis of FDOCl-1

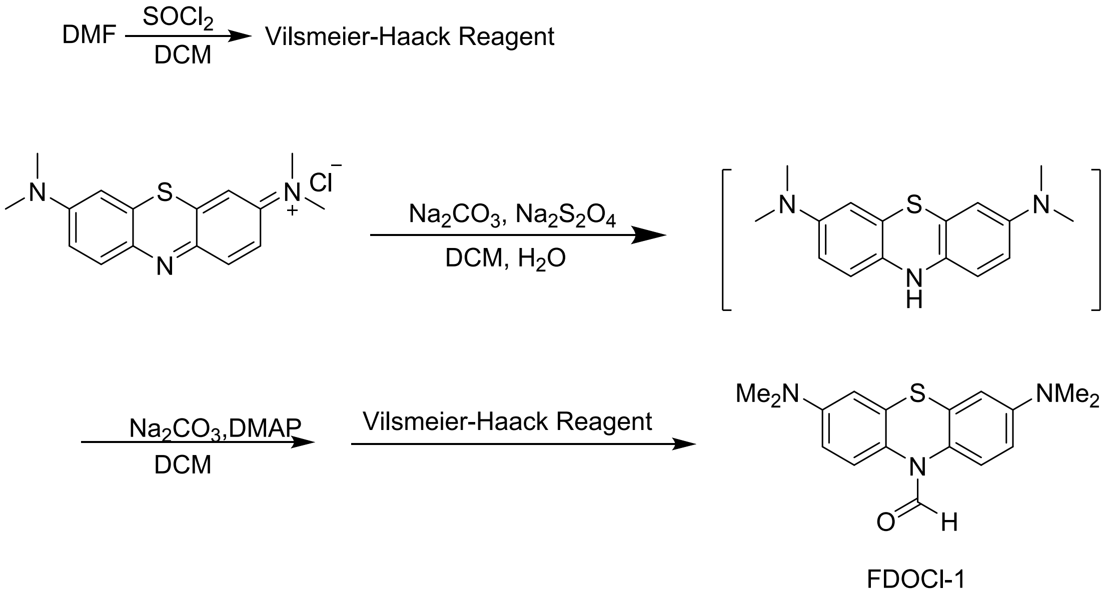

Scheme S1. The synthesis route of **FDOCl-1**.

To a stirred solution of dried DMF (0.5 mL) in 10 mL dichloromethane, thionyl chloride (1.78 g, 15 mmol, 4.0 eq) dissolved in 5 mL dichloromethane was added dropwise at room temperature. After addition the mixture was stirred at 60°C under nitrogen atmosphere for 15 minutes and evaporated on a rotary evaporator to afford the Vilsmeier-Haack Reagent.

To a solution of methylene blue (1.12 g, 3.75 mmol, 1.0 eq) in 5 mL of water, dichloromethane (10 mL) and Na_2CO_3 (1.59 g, 15.00 mmol, 4.0 eq) were added and the mixture was stirred at 40°C under nitrogen atmosphere. Sodium dithionite (2.61 g, 15.00 mmol, 4.0 eq) was dissolved in 5 mL water and injected to the solution directly using a syringe device. After addition the mixture was stirred at 40°C under nitrogen atmosphere until the solution became yellow (typically within 15-30 min). The dichloromethane layer was separated from water layer and dried with anhydrous sodium sulfate quickly. After sodium sulfate was removed by filtration, the solution was added dropwise to a mixture of Vilsmeier-Haack Reagent, DMAP (0.46 g, 3.75

mmol, 1.0 eq), Na_2CO_3 (1.19 g, 11.25 mmol, 3.0 eq) in 5 mL dichloromethane. After addition the mixture was stirred in an ice-water bath for 1 h and then at room temperature until the reaction completed as indicated by TLC analysis.

Removing the undissolved substance by filtration, the solution was poured into 200 mL of ice-water while stirring, and the resulting mixture was extracted with 3 × 100 mL portions of ethyl acetate. The combined extracts were washed with brine, dried over anhydrous sodium sulfate and evaporated on a rotary evaporator to afford a solid residue, which was purified by column chromatography (ethyl acetate/n-hexane = 1/5) to yield **FDOCl-1** as a white solid. Yield 0.53 g, 45%. M. p. 192.8 ~ 193.8°C; ^{1}H NMR (400 MHz, DMSO-d_6) δ 8.52 (s, 1H), 7.42 (d, J = 8.4 Hz, 1H), 7.29 (d, J = 8.8 Hz, 1H), 6.75 (d, J = 2.8 Hz, 1H), 6.76 - 6.66 (m, 3H), 2.90 (s, 12H). ^{13}C NMR (100 MHz, DMSO-d_6) δ 160.97, 149.11, 148.84, 130.23, 129.47, 126.64, 125.66, 124.53, 122.70, 111.21, 110.54, 110.11, 109.64, 40.19. HRMS (ESI): $[M + H]^+$ calcd for $C_{17}H_{20}N_3OS$: 314.1322; found: 314.1319.

1.2 The synthesis of FDOCl-2

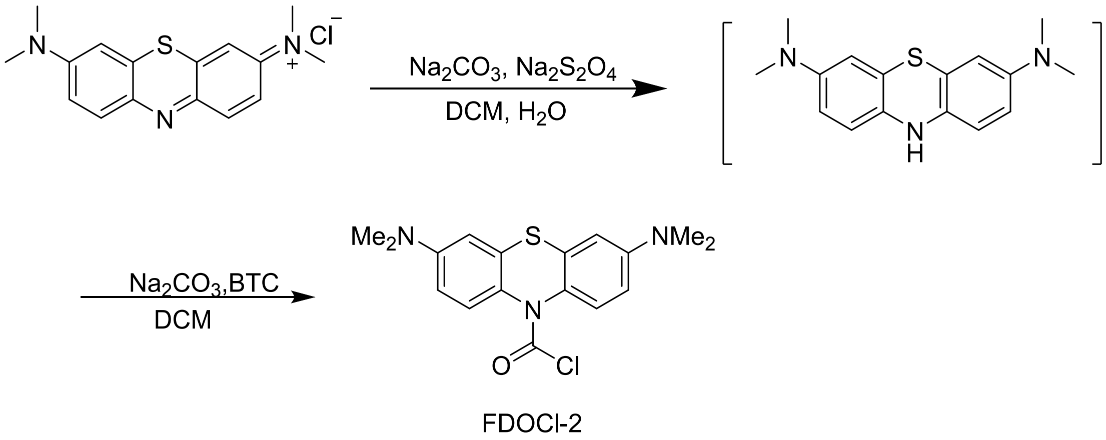

Scheme S2. The synthesis route of **FDOCl-2**.

To a solution of methylene blue (5.00 g, 15.63 mmol, 1.0 eq) in 50 mL of water, dichloromethane (25 mL) and Na_2CO_3 (6.63 g, 62.52 mmol, 4.0 eq) were added and the mixture was stirred at 40°C under nitrogen atmosphere. Sodium dithionite (10.89 g, 62.52 mmol, 4 eq) dissolved in 70 mL water was injected to the solution directly

using a syringe device. After addition the mixture was stirred at 40°C under nitrogen atmosphere until the solution became yellow (typically within 15-30 min). The mixture was cooled with an ice-water bath, to which bis(trichloromethyl)carbonate (2.78 g, 9.38 mmol, 0.6 eq) in 20 mL of dichloromethane was added dropwise. After addition the mixture was stirred for another 1 h. The solution was poured into 200 mL of ice-water while stirring, and the resulting mixture was extracted with 3 × 100 mL portions of dichloromethane. The combined extracts were washed with brine, dried over anhydrous sodium sulfate and evaporated on a rotary evaporator then purified by column chromatography (ethyl acetate/n-hexane = 1/10) to yield **FDOCl-2** as a white solid. Yield 2.83 g, 52%. ^{1}H NMR (400 MHz, DMSO-d_6) δ 7.42 (d, J = 9.2 Hz, 2H), 6.78 (d, J = 2.8 Hz, 2H), 6.70 (dd, J = 8.8, 2.8 Hz, 2H), 2.92 (s, 12H). ^{13}C NMR (100 MHz, $CDCl_3$) δ 149.97, 149.45, 134.09, 128.06, 110.85, 110.36, 77.37, 40.66. HRMS (ESI): $[M + H]^+$ calcd for $C_{17}H_{19}ClN_3OS$: 348.0932; found: 348.0927.

1.3 The synthesis of FDOCl-3

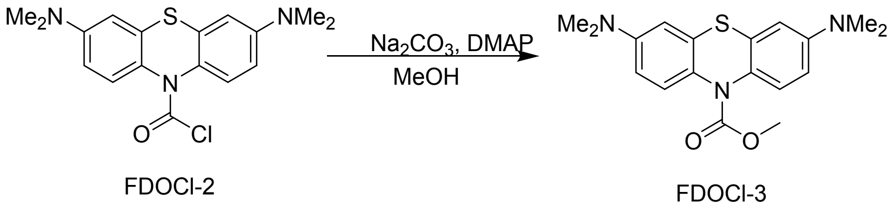

Scheme S3. The synthesis route of **FDOCl-3**.

FDOCl-2 (1.0 g, 2.87 mmol, 1.0 eq), Na_2CO_3 (0.91 g, 8.61 mmol, 3.0 eq), DMAP (0.35 g, 2.87 mmol, 1.0 eq) were dissolved in 10 mL of methanol and the resulting mixture was stirred in an ice-water bath until the reaction completed as indicated by TLC analysis which was conducted at 1 h intervals.

The reaction mixture was poured into 200 mL of ice-water while stirring, and was extracted with 3 × 100 mL portions of ethyl acetate. The combined extracts were washed with brine, dried over anhydrous sodium sulfate and evaporated on a rotary

evaporator to afford a solid residue, which was purified by column chromatography (ethyl acetate/petroleum ether = 1/5) to yield **FDOCl-3** as a white solid. Yield 0.59 g, 60%. M. p. 155.3 ~ 156.0°C. ^{1}H NMR (400 MHz, DMSO-d$_6$) δ 7.30 (d, J = 8.4 Hz, 2H), 6.68 – 6.65 (m, 4H), 3.65 (s, 3H), 2.89 (s, 12H). ^{13}C NMR (100 MHz, DMSO-d$_6$) δ 154.19, 148.60, 131.96, 127.61, 127.00, 110.94, 109.69, 53.03, 40.21. HRMS (ESI): [M + H]$^+$ calcd for $C_{18}H_{22}N_3O_2S$: 344.1427; found: 344.1434.

1.4 The synthesis of FDOCl-4

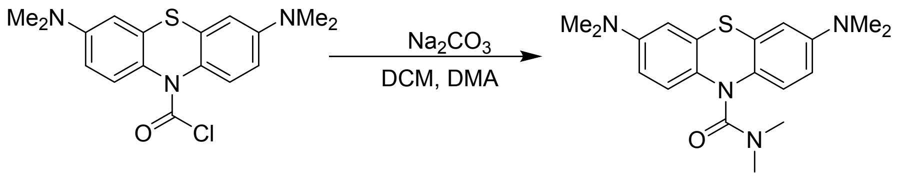

FDOCl-2

FDOCl-4

Scheme S4. The synthesis route of **FDOCl-4**.

FDOCl-2 (1.0 g, 2.87 mmol, 1.0 eq), Na_2CO_3 (0.91 g, 8.61 mmol, 3.0 eq) were dissolved in 10 mL of dichloromethane, the resulting mixture was stirred in an ice-water bath. Dimethylamine (0.52 g, 11.48 mmol, 4.0 eq) in 5 mL of dichloromethane was added dropwise. After addition, the mixture was stirred at room temperature until the reaction completed as indicated by TLC analysis which was conducted at 1 h intervals.

The reaction mixture was poured into 200 mL of ice-water while stirring, and the resulting mixture was extracted with 3 × 150 mL portions of ethyl acetate. The combined extracts were washed with brine, dried over anhydrous sodium sulfate and evaporated on a rotary evaporator to afford an oily residue, which was purified by column chromatography (ethyl acetate/petroleum ether = 1/5) to yield **FDOCl-4** as a white solid. Yield 0.36 g, 35%. M. p. 154.7 ~ 155.3°C. ^{1}H NMR (400 MHz, DMSO-d$_6$) δ 7.42 (d, J = 8.8 Hz, 2H), 6.68 (d, J = 2.8 Hz, 2H), 6.63 (dd, J = 8.8, 2.8 Hz, 2H), 2.86 (s, 12H), 2.64 (s, 6H). ^{13}C NMR (101 MHz, DMSO-d$_6$) δ 157.98, 147.78, 131.11, 129.81, 122.85, 111.40, 110.44, 40.30, 37.49. HRMS (ESI): [M + H]$^+$ calcd for

$C_{19}H_{25}N_4OS$: 357.1744; found: 357.1757.

2 Additional tables

Table S1 Some recently published fluorescet probes based on different detection mechanism for HOCl imaging[a]

Detection mechanisms	Probes	Absorbance change	Detection limit/nM[b]	Sensitivity[c]	Selectivity[d]	In vivo application
Oxidation of substituted phenol analogues	HKOCl-1[1]	--	--	1079-fold (1.0 equiv)	19-fold (10 μM HOCl / 10 μM $ONOO^-$)	--
	HKOCl-2b[2]	2-fold decrease (10 μM probe / 20 μM HOCl)	18	908-fold (2.0 equiv)	>20-fold (10 μM HOCl / 100 μM $ONOO^-$)	--
	HKOCl-3[3]	Red shift <50nm	0.33	358-fold (1.0 equiv)	83-fold (100 μM HOCl / 100 μM $ONOO^-$)	Fluorescent imaging of HOCl in live zebrafish embryos
	FCN2[4]	--	6.68	1643.4-fold (3.3 equiv)	36-fold over $ONOO^-$ (5 μM HOCl / 50 μM $ONOO^-$)	Fluorescent imaging of accumulated HOCl in specific organelles using a zebrafish model
Deoximation of luminescent oximes	Phenanthroimidazole-oxime[5]	Red shift > 20nm	--	9.8-fold (ratio, I509/I439, 30 equiv)	>5-fold (ratio, I509/I439, 300 μM HOCl / 300 μM H_2O_2)	--
	Flu-1[6]	--	--	61-fold (20 equiv)	>20-fold (20 μM HOCl / 40 μM H_2O_2)	--
	BOD-OXINE[7]	--	17.7	9-fold (50 equiv)	>5-fold (100 μM HOCl / 100 μM	--

					$ONOO^{-}$)	
	HCH[8]	Blue shift 6 nm	58	14.1-fold (25 equiv)	>10-fold (250 μM HOCl / 250 μM •OH)	--
Chlorination of thioesters or amides	Cou-Rho-HOCl[9]	> 200-fold increase (5 μM probe / 30 equiv HOCl)	52	> 50-fold (ratio, I594/I473, 30 equiv)	>10-fold (ratio, I594/I473, 5 μM HOCl / 5 equiv •OH)	--
	Ru-Fc[10]	--	38.6	60-fold (6.0 equiv)	>20-fold (60 μM HOCl / 60 μM •OH)	Fluorescent imaging of HOCl in daphnia magna and zebrafish
	R19-S[11, 12]	--	--	> 50-fold (1.0 equiv)	>20-fold (10 μM HOCl / 200 μM •OH)	Fluorescent imaging of intestinal HOCl production in the drosophila system
	FBS[13]	>5-fold increase (2 μM probe / 20 μM HOCl)	200	> 50-fold (9.5 equiv)	>20-fold (19 μM HOCl / 22 μM $ONOO^{-}$)	Fluorescent imaging of intestinal HOCl production in the drosophila system
Oxidation of thioethers to sulfonates or selenides to selenoxides	HySOx[14]	>10-fold increase (5 μM probe / 5 μM HOCl)	--	> 50-fold (2.5 equiv)	10-fold over •OH (5 μM HOCl /100 μM)	--
	MMSiR[15]	>10-fold increase (5 μM probe / 5 μM HOCl)	--	> 50-fold (1.0 equiv)	>20-fold (5 μM HOCl / 5 μM $ONOO^{-}$)	Fluorescent imaging of HOCl generation in a PMA induced mouse peritonitis model
	MITO-TP[16]	--	17.2	634-fold (20.0 equiv)	>50-fold (100 μM HOCl / 100 μM	Fluorescent imaging

					ONOO$^-$)	of LPS-dependent HOCl generation in inflammation tissues
	LYSO-TP[16]	--	19.6	610-fold (20.0 equiv)	>50-fold (100 μM HOCl / 100 μM ONOO$^-$)	Fluorescent imaging of LPS-dependent HOCl generation in inflammation tissues
	CM1[17]	>5-fold increase (4 μM probe / 100 μM HOCl)	10	> 50-fold (7.0 equiv)	>20-fold (100 μM HOCl / 200 μM ONOO$^-$)	--
	PIS[18]	Blue shift 42 nm	71	> 5-fold (5.0 equiv)	>5-fold (10 μM HOCl / 200 μM $^•$OH)	Fluorescent imaging of HOCl using fresh rat hippocampal slice
	SeCy7[19]	--	310	19.4-fold (2.0 equiv)	4.8-fold over $^1O_2$ (30 μM HOCl / 600 μM $^•$OH)	Fluorescent imaging of LPS and PMA induced HOCl production in living mice
	NI-Se[20]	--	586	> 10-fold (10.0 equiv)	> 10-fold (100 μM HOCl / 200 μM ONOO$^-$)	Fluorescent imaging of LPS induced HOCl production in living mice
	FO-PSe[21]	--	350	> 10-fold (1.0 equiv)	> 10-fold (10 μM HOCl / 100 μM ONOO$^-$)	Fluorescent imaging of HOCl in zebrafish and mice

	$PFOBT_{36}SeT$ BT_5 Pdots[22]	Blue shift 42 nm	500	40-fold (ratio, I540/I680, 250 µM HOCl)	>6-fold (100 µM HOCl / 1 mM •OH)	Fluorescent imaging of LPS-induced HOCl generation in peritonitis of living mice
Cleavage of carbon-carbon double bonds	9-AEF[23]	>3-fold decrease (100 µM probe / 500 µM HOCl)	300	160-fold (1.0 equiv)	>100-fold •OH (100 µM HOCl /100 µM)	--
	hCy3-csUCNP:Nd[24]	>10-fold decrease (probe 0.1 mg mL^{-1} / 20 µM HOCl at 550 nm)	500	>5-fold probe 0.1 mg mL^{-1} / 20 µM at 550 nm	>5-fold •OH (20 µM HOCl /20 µM •OH)	Fluorescent imaging of λ-carrageenan induced HOCl generation in mouse model of arthritis
Deformylation reaction (this work)	FDOCl-1	577-fold increase (10 µM probe / 25 µM HOCl)	UV: 3.98 nM; FL: 2.62 nM	2068-fold (2.5 equiv)	313-fold •OH (25 µM HOCl /100 µM •OH)	Fluorescent and naked eye imaging of λ-carrageenan induced HOCl generation in mouse model of arthritis

[a] If no actual data were provided in the references, data were estimated according to the original published figures.

[b] Reported detection limit provided in the references.

[c] Fluorescence intensity or emission ratio changes before and after treated with HOCl.

[d] Fluorescence intensity or emission ratio changes before and after treated with HOCl.

Table S2 Crystal data and structure refinement details for **FDOCl-1** and **FDOCl-4**

	**FDOCl-1**	**FDOCl-4**
Identification code	mo_51231ba	mo_70223a
Empirical formula	C17 H19 N3 O S	C19 H24 N4 O S
Formula weight	313.41	356.48
Temperature	296(2) K	223(2) K
Wavelength	0.71073 Å	0.71073 Å
Crystal system	Monoclinic	Monoclinic
Space group	P 21/c	$P2_1/n$
Unit cell dimensions	a = 16.166(4) Å	a = 5.9414(11) Å
	b = 8.073(2) Å	b = 15.879(3) Å
	c = 12.176(3) Å	c = 19.207(4) Å
Volume	1566.1(7) $Å^3$	1807.4(6) $Å^3$
Z	4	4
Density (calculated)	1.329 Mg/m^3	1.310 Mg/m^3
Absorption coefficient	0.212 mm^{-1}	0.194 mm^{-1}
F(000)	664	760
Crystal size	0.480 x 0.080 x 0.040 mm^3	0.380 x 0.280 x 0.080 mm^3
Theta range for data collection	2.557 to 27.411°.	1.666 to 27.613°.
Index ranges	-20<=h<=20, -10<=k<=8, -15<=l<=15	-7<=h<=7, -20<=k<=20, -15<=l<=24
Reflections collected	10950	13232
Independent reflections	3538 [R(int) = 0.0440]	4176 [R(int) = 0.0532]
Completeness to theta = 25.242°	99.4 %	99.5 %
Absorption correction	Semi-empirical from equivalents	Semi-empirical from equivalents
Max. and min. transmission	0.746 and 0.690	0.956 and 0.860
Refinement method	Full-matrix least-squares on F^2	Full-matrix least-squares on F^2
Data / restraints / parameters	3538 / 0 / 203	4176 / 0 / 233
Goodness-of-fit on F^2	1.005	1.010
Final R indices [I>2sigma(I)]	R1 = 0.0433, wR2 = 0.1016	R1 = 0.0461, wR2 = 0.0991
R indices (all data)	R1 = 0.0836, wR2 = 0.1188	R1 = 0.0791, wR2 = 0.1139
Extinction coefficient	n/a	0.0098(12)
Largest diff. peak and hole	0.191 and -0.202 e.$Å^{-3}$	0.257 and -0.236 e.$Å^{-3}$
Max. and min. transmission	0.746 and 0.690	--

Table S3 Fluorescence responses of **FDOCl-1** (10 μM) toward different concentration of HOCl

Concentration (μM)	Fluorescence Intensity
0	17
1	1324
5	9759
10	22090
25	35150

Table S4 Fluorescence responses of **FDOCl-1** (10 μM) toward various ROS/RNS

	Concentration (μM)	Fluorescence Intensity
H_2O_2	25	<35
	50	<35
	100	<35
O_2^-	25	<35
	50	<35
	100	<35
t-BuOOH	25	<35
	50	<35
	100	<35
$^{\bullet}$OH	25	<35
	50	39.5
	100	112.3
NO	25	<35
	50	<35
	100	<35
$ONOO^-$	25	<35
	50	<35
	100	<35
ROO$^{\bullet}$	25	<35
	50	<35
	100	<35
t-BuOO$^{\bullet}$	25	37.25
	50	62.25
	100	103.5

Table S5 Absorbance changes of **FDOCl-1** (10 μM) toward different concentration of HOCl

Concentration (μM)	Absorbance
0	0.001
1	0.118
5	0.22
10	0.322
25	0.577
35	0.696

Table S6 Absorbance changes of **FDOCl-1** (10 μM) toward various ROS/RNS

	Concentration (μM)	Absorbance
H_2O_2	25	<0.005
	50	<0.005
	100	<0.005
O_2^-	25	<0.005
	50	<0.005
	100	<0.005
t-BuOOH	25	<0.005
	50	<0.005
	100	<0.005
$^{\bullet}OH$	25	<0.005
	50	<0.005
	100	0.013
$NO^{\bullet}$	25	<0.005
	50	<0.005
	100	<0.005
$ONOO^-$	25	<0.005
	50	<0.005
	100	<0.005
$ROO^{\bullet}$	25	<0.005
	50	<0.005
	100	<0.005
t-BuOO$^{\bullet}$	25	<0.005
	50	0.006
	100	0.01

Table S7 Other Photophysical Parameters of **FDOCl-1** before and after treated with HOCl[a]

	ε^{b} (M^{-1} cm^{-1})	Φ^{c}	Brightness[d] (M^{-1} cm^{-1})
**FDOCl-1** (10 μM)	100	-	-
**FDOCl-1** (10 μM) + HOCl (25 μM)[e]	57,700	0.02	1154

[a] The data was recorded in sodium phosphate buffer (10 mM, pH 7.2, 0.1% EtOH)

[b] Molar absorption coefficient at 664 nm

[c] Φ: absolute fluorescence quantum yield

[d] Brightness = $\varepsilon \times \Phi$, at 664 nm[25]

[e] The data was recorded after 5 min

3. Additional images and figures

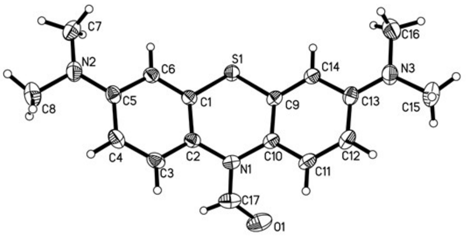

FDOCl-1

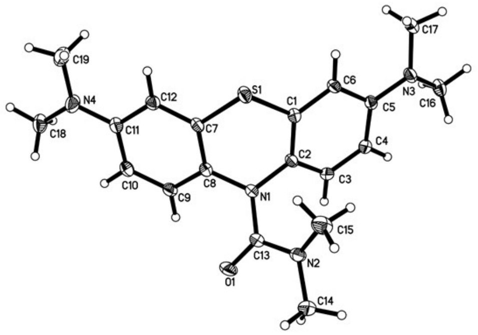

FDOCl-4

Fig. S1 Crystal structure of **FDOCl-1** and **FDOCl-4**

**Acquisition Parameter**					
Source Type	ESI	Ion Polarity	Positive	Set Nebulizer	1.0 Bar
Focus	Not active			Set Dry Heater	180 ℃
Scan Begin	50 m/z	Set Capillary	2500 V	Set Dry Gas	6.0 l/min
Scan End	1000 m/z	Set End Plate Offset	-500 V	Set Divert Valve	Waste

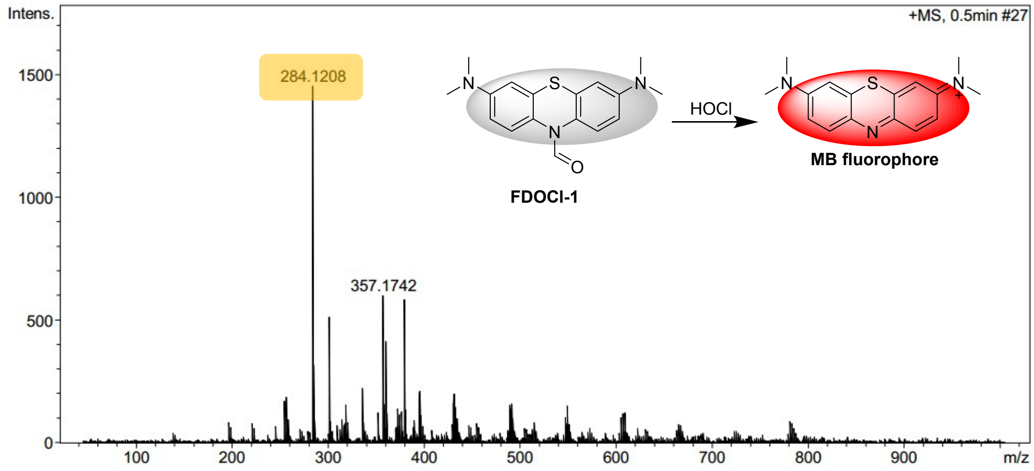

Fig. S2 HRMS analysis of **FDOCl-1** after reacted with HOCl

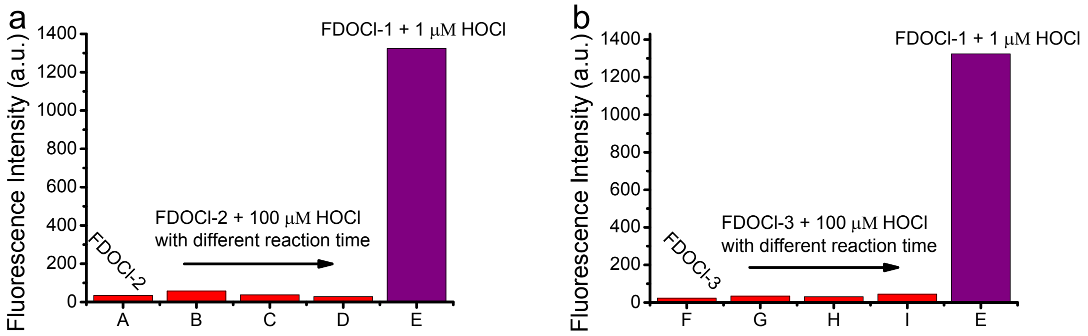

Fig. S3 Fluorescence intensity change at 686 nm of (a) **FDOCl-2** and (b) **FDOCl-3** after adding 100 μM HOCl with different reaction time compared with **FDOCl-1** (A: **FDOCl-2** only; from B to D: **FDOCl-2** + 100 μM HOCl for 30 min, 60 min, 120 min; F: **FDOCl-3** only; from G to I: **FDOCl-3** + 100 μM HOCl for 30 min, 60 min, 120 min; E: **FDOCl-1** + 1 μM HOCl; probes using 10 μM; sodium phosphate buffer (PBS), 10 mM, pH 7.2, 0.1% EtOH; λ_{ex} = 620 nm)

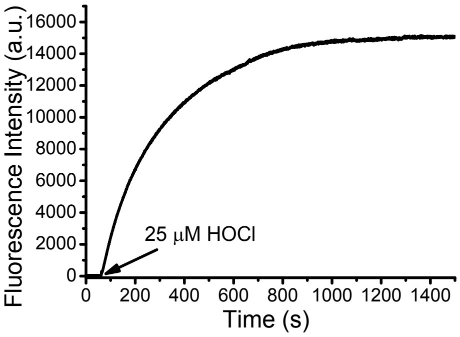

Fig. S4 Fluorescence intensity changes at 684 nm of **FDOCl-4** (10 μM) after adding HOCl (25 μM) with different reaction time (PBS, 10 mM, pH 7.2, 0.1% EtOH; λ_{ex} = 620 nm).

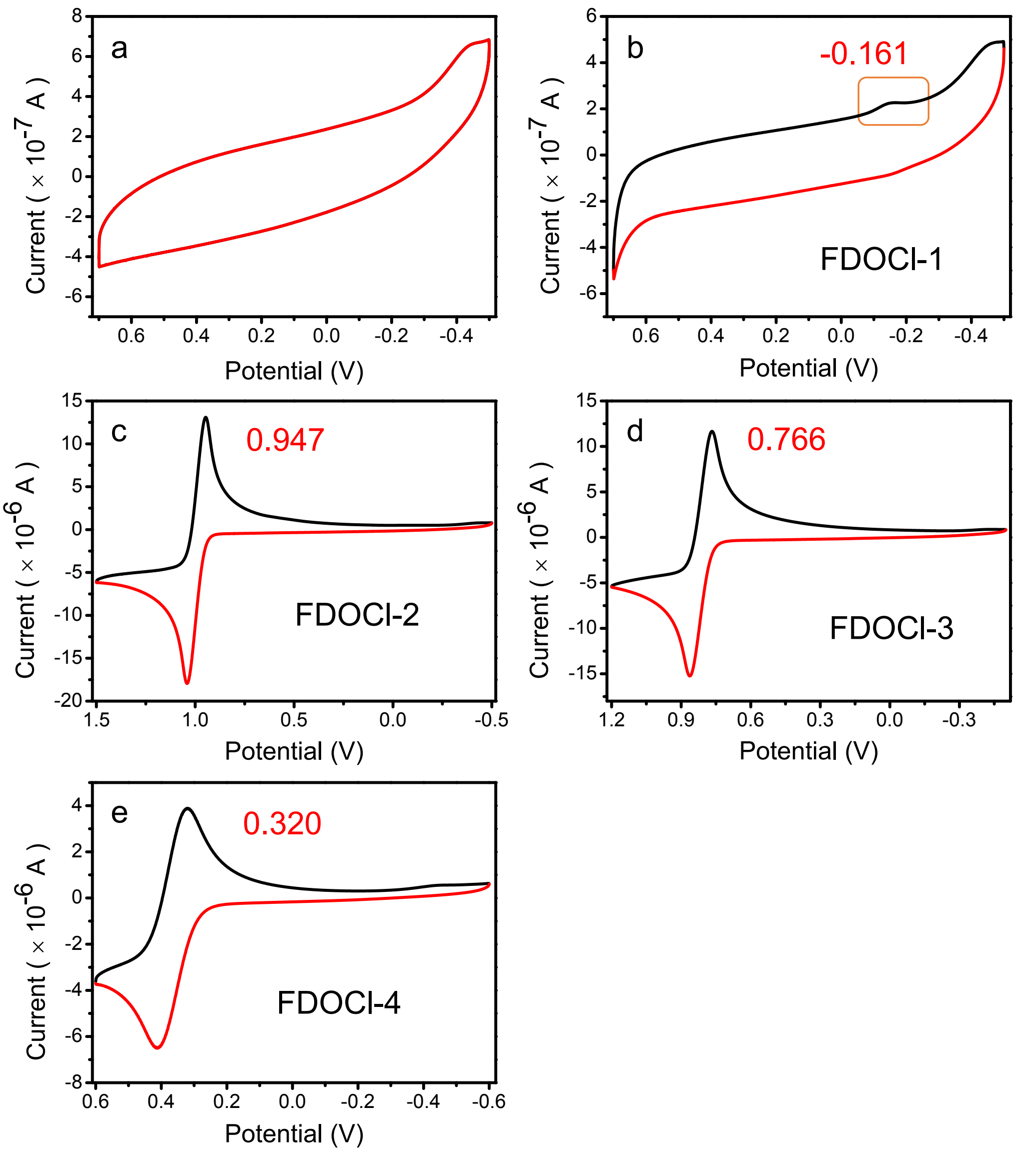

Fig. S5 Cyclic voltammograms of (a) dichloromethane (DCM), (b) **FDOCl-1**, (c) **FDOCl-2**, (d) **FDOCl-3** and (e) **FDOCl-4** in DCM medium. (Electrolyte: 0.1 M TBAPF6. Scan rate = 0.05V/s; initial scanning direction: from positive potential to negative potential; concentration c = 1 mM for each compounds)

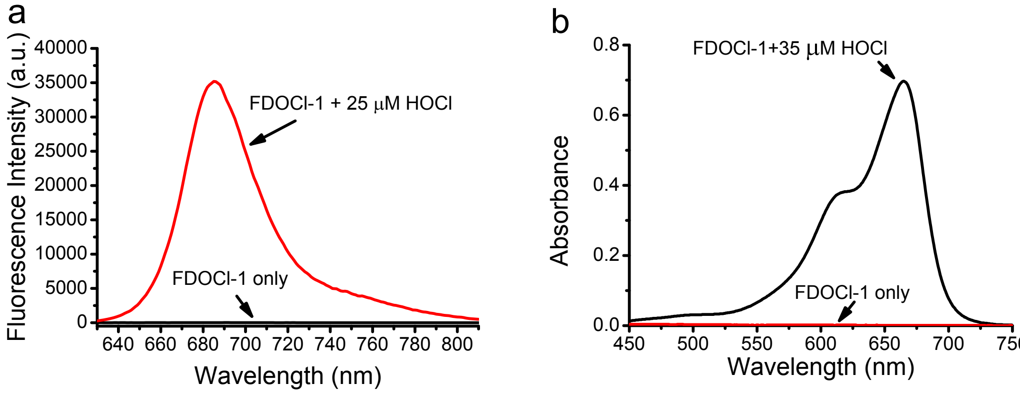

Fig. S6 (a) Fluorescence spectra of **FDOCl-1** (10 μM) before/after adding 25 μM HOCl with an excitation at 620 nm. (b) Absorption spectra of **FDOCl-1** (10 μM) before/after adding 35 μM HOCl. (The data was recorded after 5 min in PBS (10 mM, pH 7.2, 0.1% EtOH); λ_{ex} = 620 nm).

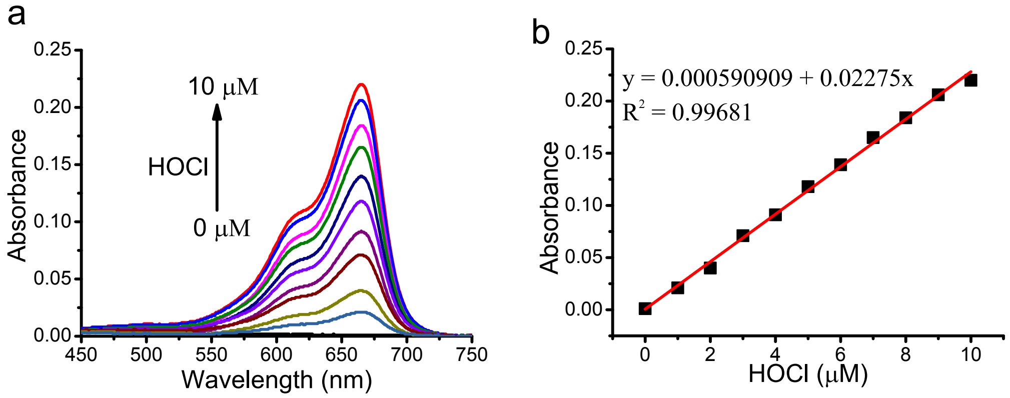

Fig. S7 (a) Absorption spectra and (b) absorbance changes at 664 nm of **FDOCl-1** (10 μM) with increasing concentrations of HOCl. (The data was recorded after 5 min in PBS (10 mM, pH 7.2, 0.1% EtOH))

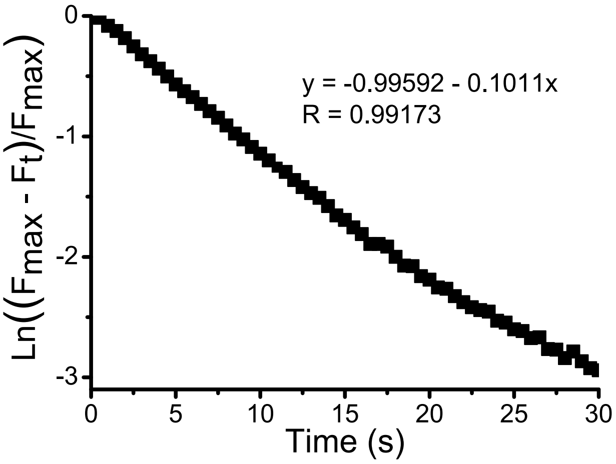

Fig. S8 Pseudo-first-order kinetic plot of the reaction of 10 μM **FDOCl-1** to 25 μM HOCl (PBS, 10 mM, pH 7.2, 0.1% EtOH; λex = 620 nm). Slop = -0.1011.

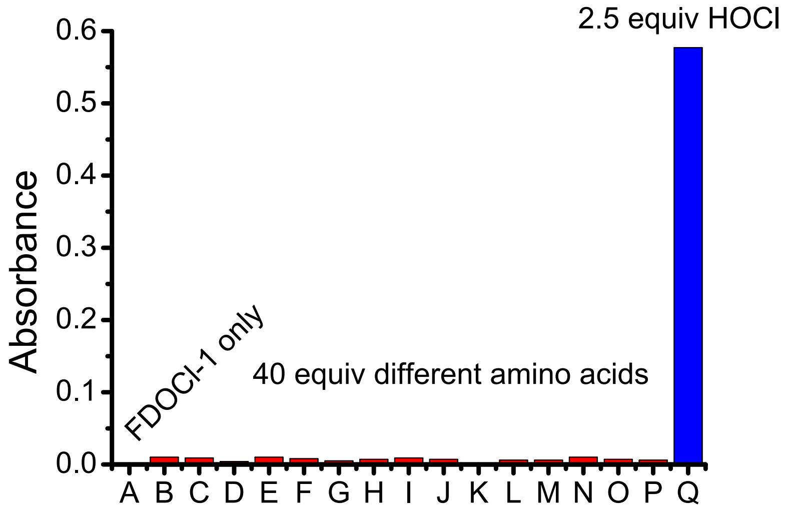

Fig. S9 Absorbance at 664 nm of **FDOCl-1** (10 μM) after adding various amino acids. From A to Q: Blank, Leu, Pro, Gly, Gln, Glu, Met, Lys, Trp, Ser, Thr, Asp, Ile, Val, His, Ala, HOCl. (The data was recorded after 5 min in PBS (10 mM, pH 7.2, 0.1% EtOH))

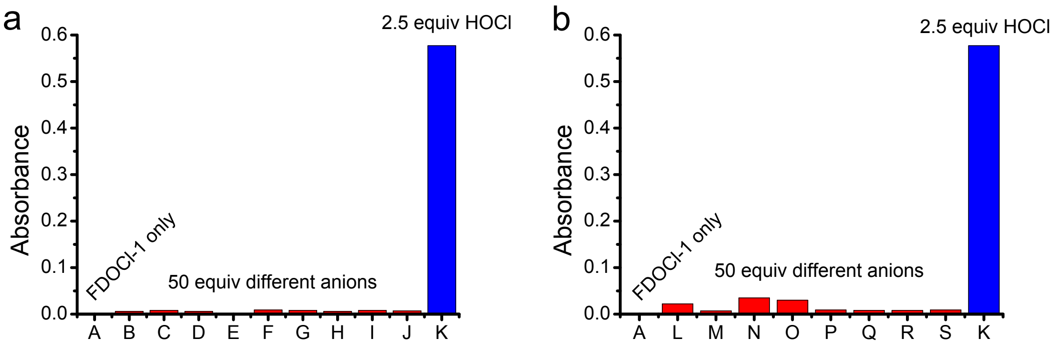

Fig. S10 (a and b) Absorbance at 664 nm of **FDOCl-1** (10 μM) after adding various anions/cations. From A to K: Blank, CH_3COO^-, CO_3^{2-}, SO_4^{2-}, Cl^-, ClO_4^-, F^-, I^-, NO_2^-, $S_2O_3^{2-}$, HOCl; from L to S: Al^{3+}, Ca^{2+}, Cu^{2+}, Fe^{3+}, K^+, Mg^{2+}, NH_4^+, Ni^+. (The data was recorded after 5 min in PBS (10 mM, pH 7.2, 0.1% EtOH))

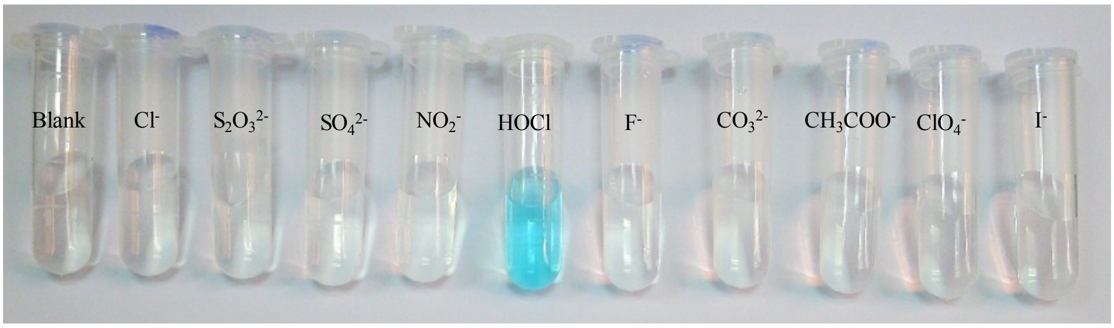

Fig. S11 Colour changes of **FDOCl-1** (10 μM) after adding different anions. (PBS 10 mM, pH 7.2, 0.1% EtOH)

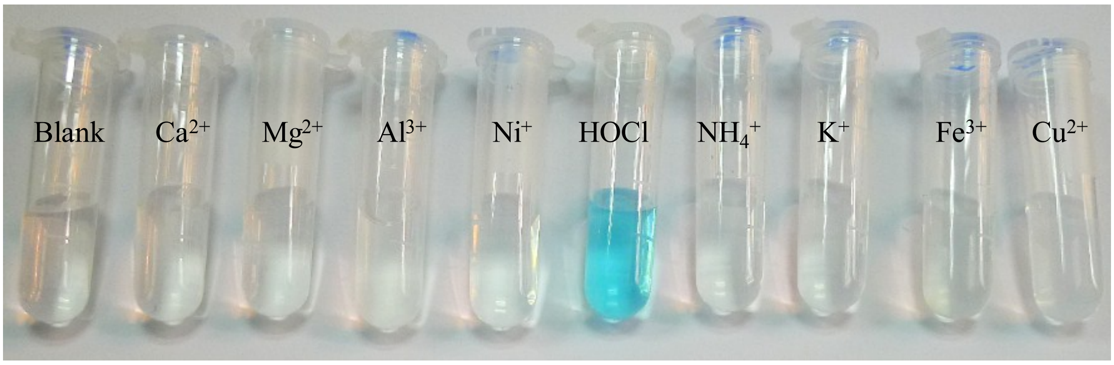

Fig. S12 Colour changes of **FDOCl-1** (10 μM) after adding different cations. (PBS, 10 mM, pH 7.2, 0.1% EtOH)

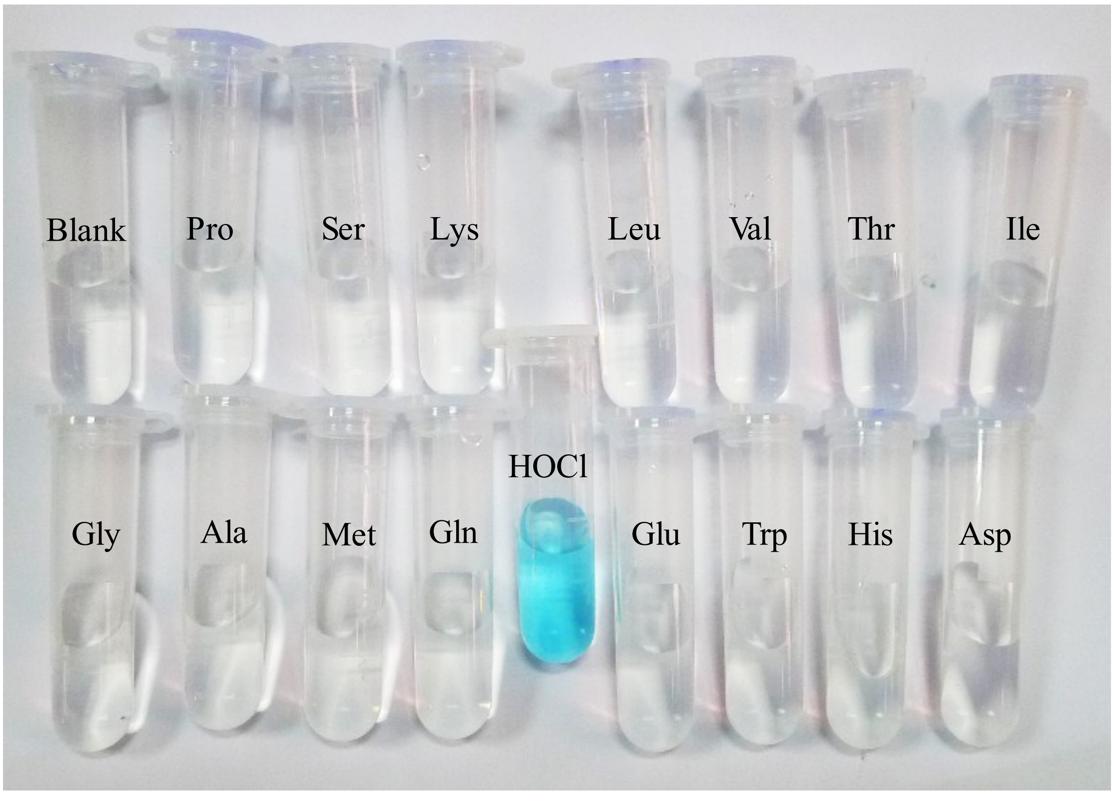

Fig. S13 Colour changes of **FDOCl-1** (10 μM) after adding various amino acids. (PBS, 10 mM, pH 7.2, 0.1% EtOH)

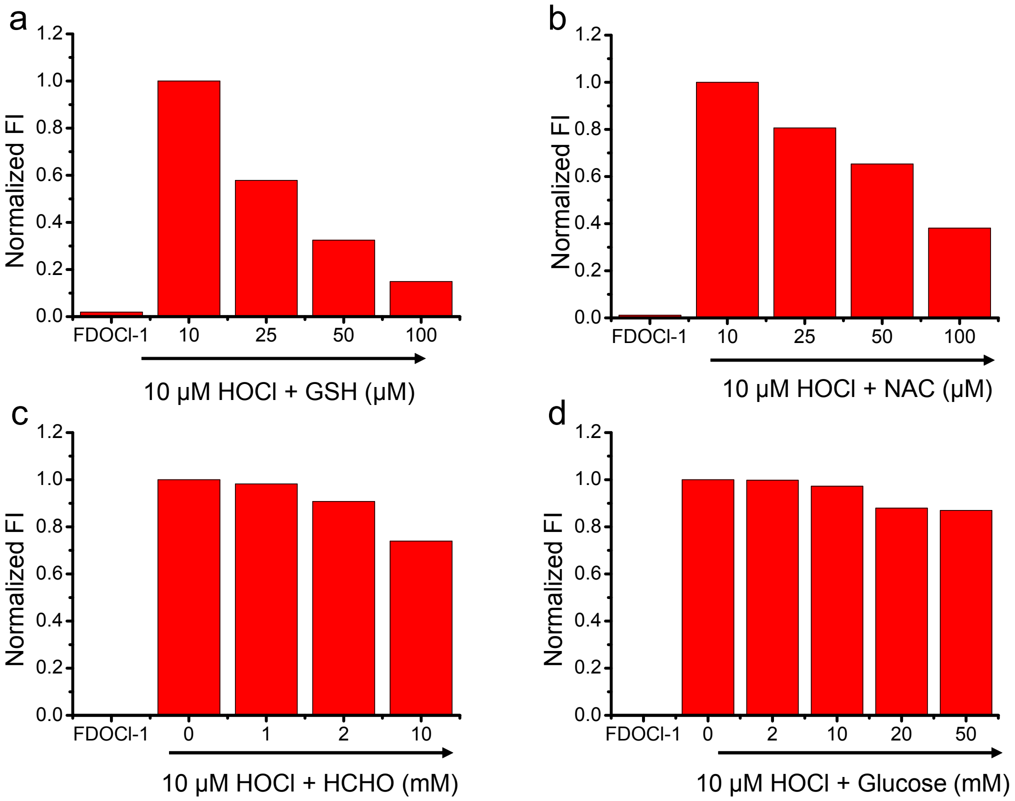

Fig. S14 Fluorescence intensity of **FDOCl-1** (10 μM) at 686 nm toward HOCl (10 μM) in the present of sulfhydryl compounds (a) GSH; (B) NAC and aldehydes (c)

HCHO; (d) Glucose. (The data was recorded after 5 min in sodium phosphate buffer (10 mM, pH 7.2, 0.1% EtOH); $\lambda_{ex} = 620$ nm)

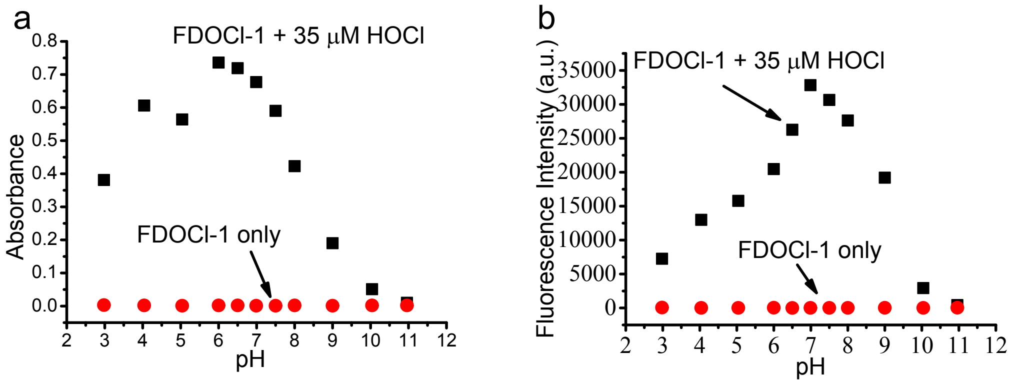

Fig. S15 (a) Fluorescence intensity at 686 nm and (b) absorption strength at 664 nm of **FDOCl-1** (10 μM) changes with different pH before and after treated with 35 μM HOCl. (The data was recorded after 5 min in PBS (10 mM, 0.1% EtOH); $\lambda_{ex} = 620$ nm)

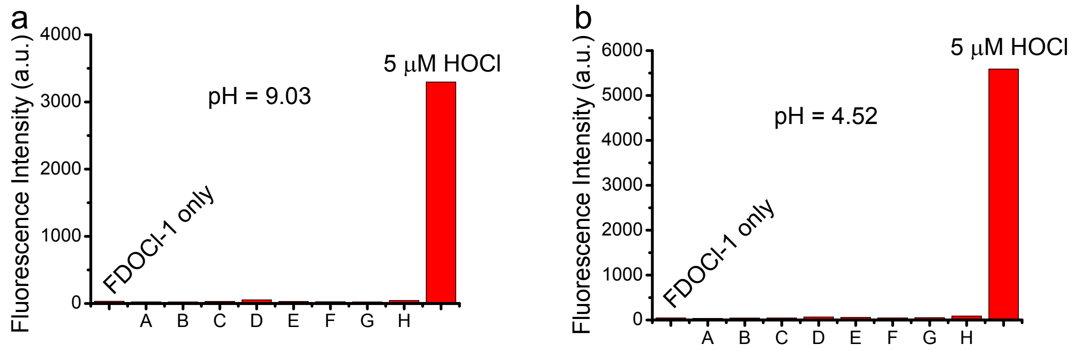

Fig. S16 Fluorescence intensity of **FDOCl-1** (10 μM) at 686 nm after adding various ROS/RNS (50 μM) in the pH value of (a) 9.03 and (b) 4.52. From A to H: H_2O_2, O_2^-, NO, •OH, ONOO⁻, ROO•, t-BuOOH, t-BuOO•. (The data was recorded after 5 min in PBS (10 mM, 0.1% EtOH); $\lambda_{ex} = 620$ nm)

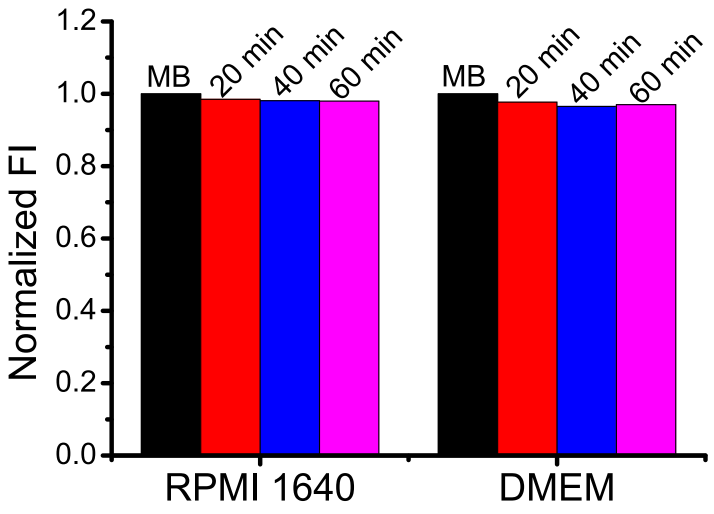

Fig. S17 Fluorescence intensity changes at 686 nm of methylene blue (10 μM), before (black)/after adding 200 μM HOCl for different time (red: 20 min; blue: 40 min; magenta: 60 min) in Dulbecco's modified essential medium (RPMI 1640) and Dulbecco's Modified Eagle's medium (DMEM). (λ_{ex} = 620 nm)

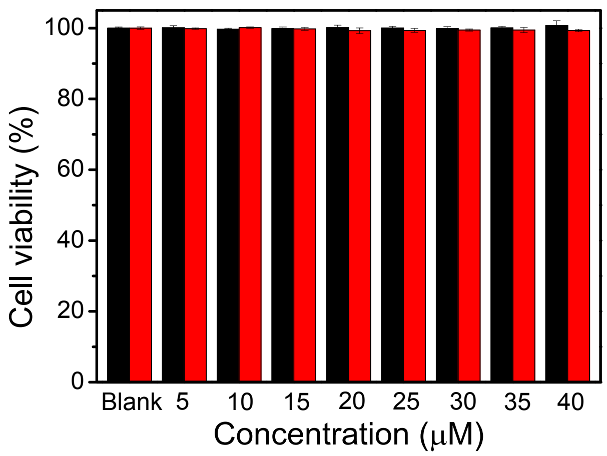

Fig. S18 The cell viability of **FDOCl-1** at different concentration (0, 5, 10, 15, 20, 25, 30, 35, 40 μM) in RAW 264.7 macrophages cells for 6 h (black) and 12 h (red) measured by MTT assay.

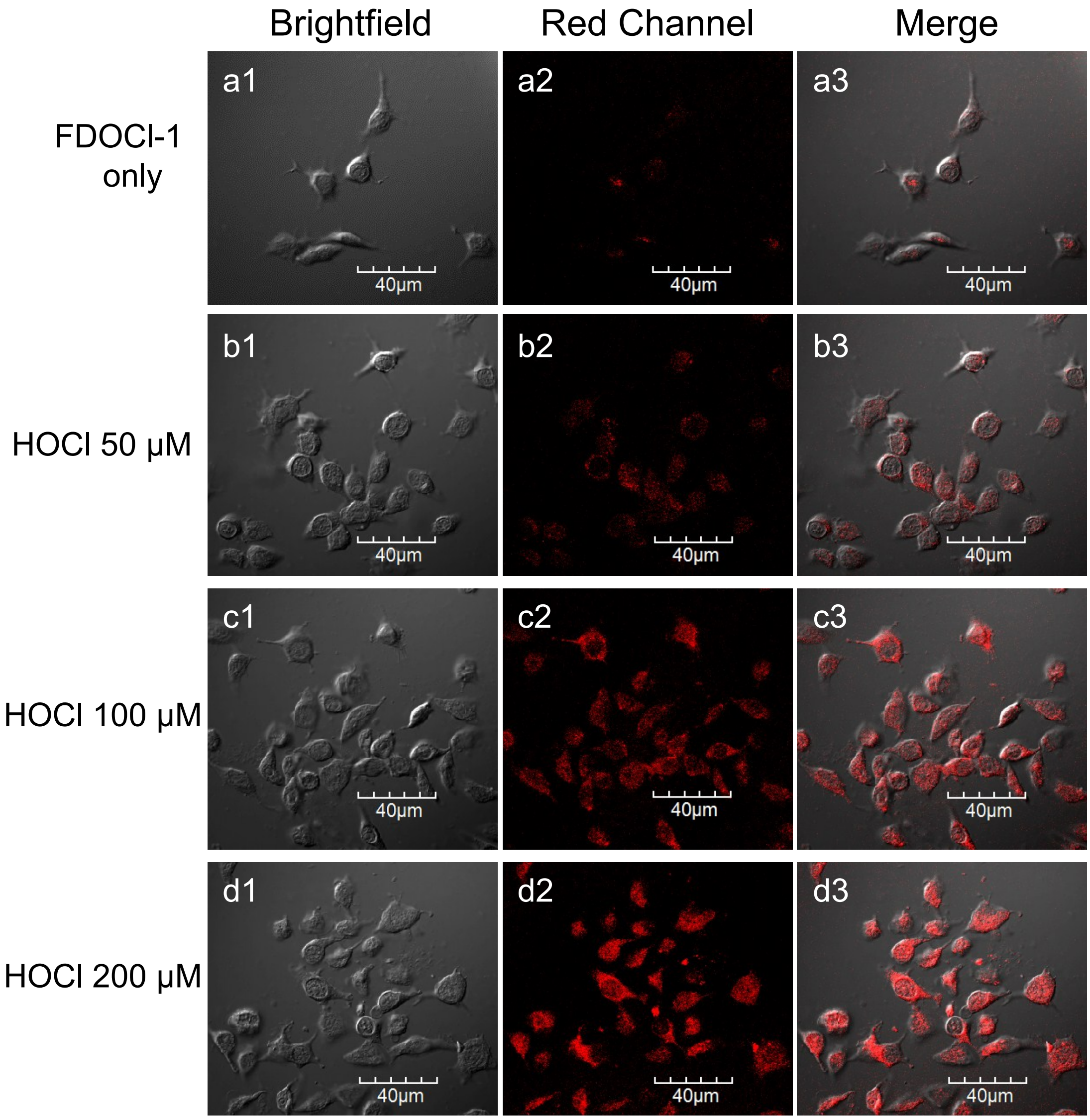

Fig. S19 GLSM images of live RAW 264.7 macrophages. (a1, a2, a3) Cells were incubated with **FDOCl-1** (10 μM) for 60 min and washed by PBS buffer. Cells were preincubated with **FDOCl-1** (10 μM) for 60 min, washed by PBS buffer and incubated with HOCl (50 μM: b1, b2, b3; 100 μM: c1, c2, c3; 200 μM: d1, d2, d3) for 10 min. (CLSM imaging was performed on an Olympus FV1000 confocal scanning system with a 60×immersion objective lens. Red channel: 700 ± 50 nm, λex = 633 nm)

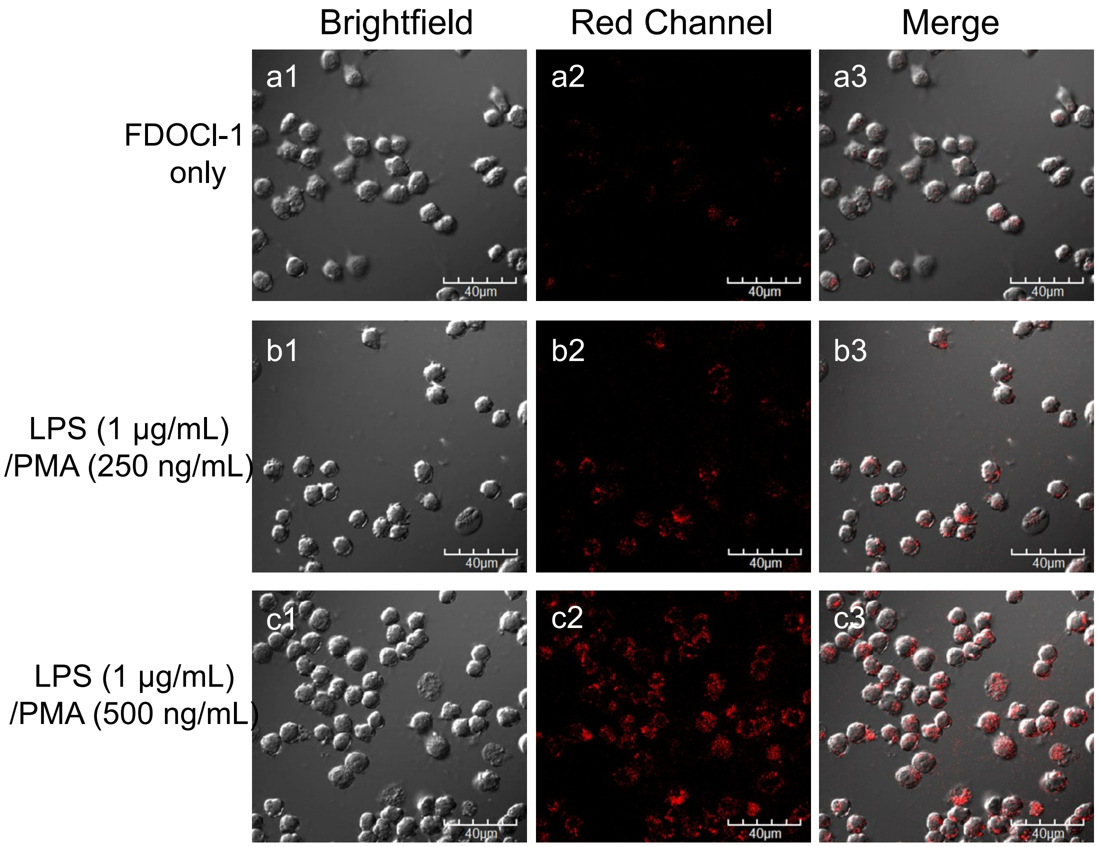

Fig. S20 GLSM images of live RAW 264.7 macrophages. (a1, a2, a3) Cells were incubated with **FDOCl-1** (10 μM) for 60 min and washed by PBS buffer. Cells were preincubated with probes, washed by PBS buffer and stimulated with LPS (1 μg/mL)/PMA (250 ng/mL: b1, b2, b3; 500 ng/mL: c1, c2, c3) for 1 h. (CLSM imaging was performed on an Olympus FV1000 confocal scanning system with a 60×immersion objective lens. Red channel: 700 ± 50 nm, λex = 633 nm)

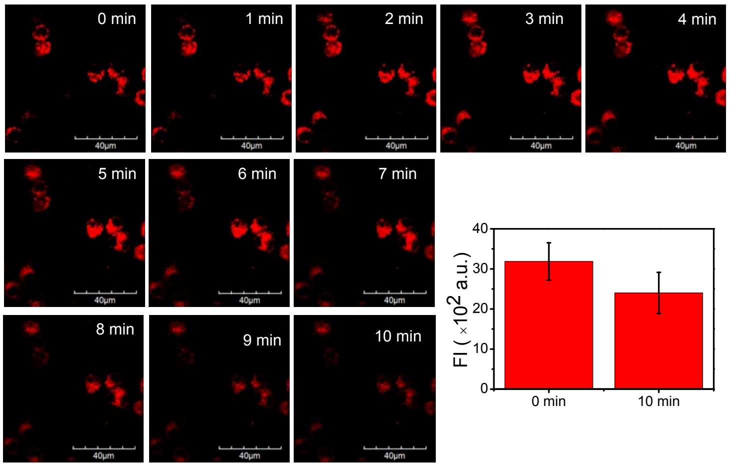

Fig. S21 Photostability of MB in live cells. Fluorescence images were taken by time-sequential scanning of live RAW 264.7 macrophages preincubated with 5 μM MB for 30 min. (CLSM imaging was performed on an Olympus FV1000 confocal scanning

system with a 60×immersion objective lens. Red channel: 700 ± 50 nm, λex = 633 nm)

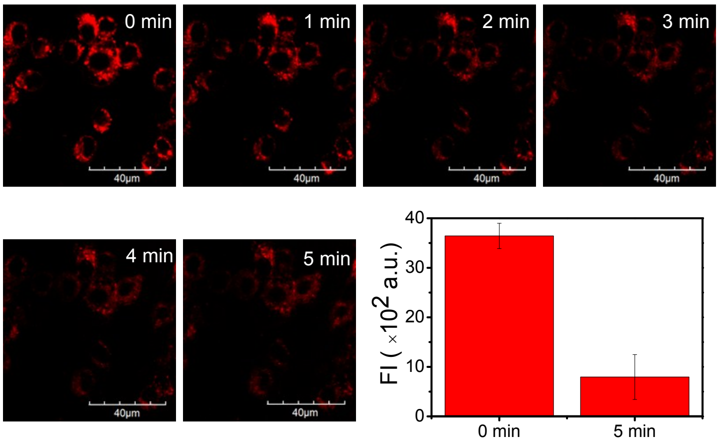

Fig. S22 Photostability of Cy5 in live cells. Fluorescence images were taken by time-sequential scanning of live RAW 264.7 macrophages preincubated with 5 μM Cy5 for 30 min. (CLSM imaging was performed on an Olympus FV1000 confocal scanning system with a 60×immersion objective lens. Red channel: 700 ± 50 nm, λex = 633 nm)

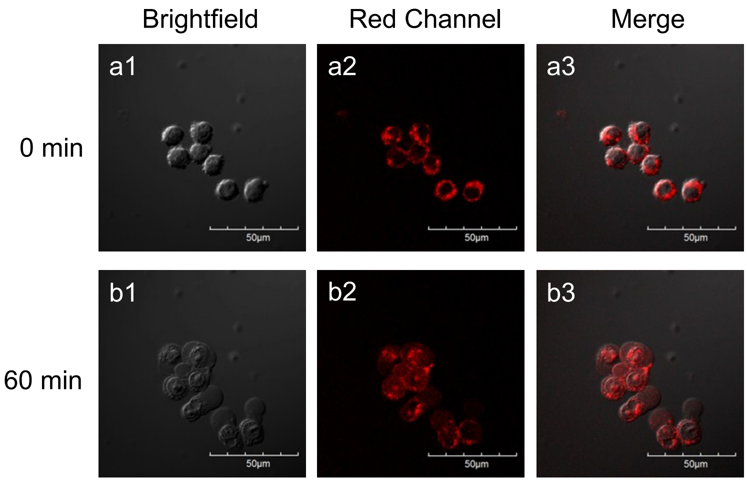

Fig. S23 GLSM images of live RAW 264.7 macrophages preincubated with MB (10 μM) for 30 min, washed by PBS buffer immediately (a1, a2, a3) and after 60 min (b1, b2, b3). (CLSM imaging was performed on an Olympus FV1000 confocal scanning system with a 60×immersion objective lens. Red channel: 700 ± 50 nm, λex = 633 nm)

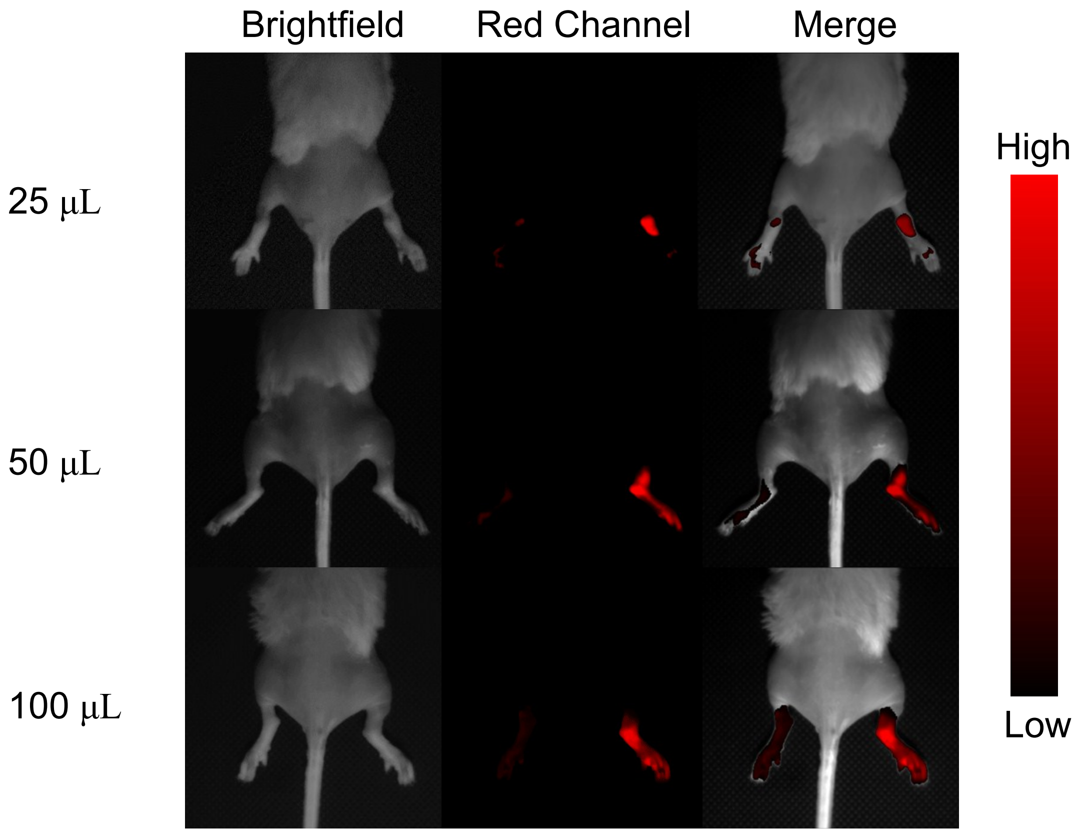

Fig. S24 *In vivo* fluorescence imaging of the arthritis model.(arthritis model induced by injecting of different volume of λ-carrageenan, 5 mg/mL, in PBS into the right tibiotarsal ankles; the left tibiotarsal ankles as the control group without jnjection of λ-carrageenan; the fluorescence signal was collected at λem = 720 ± 60 nm under excitation with 635 nm CW laser, power density is 0.3 mW cm^{-2}; **FDOCl-1**, 100 μL, 1 mM)

4 NMR and HRMS spectra

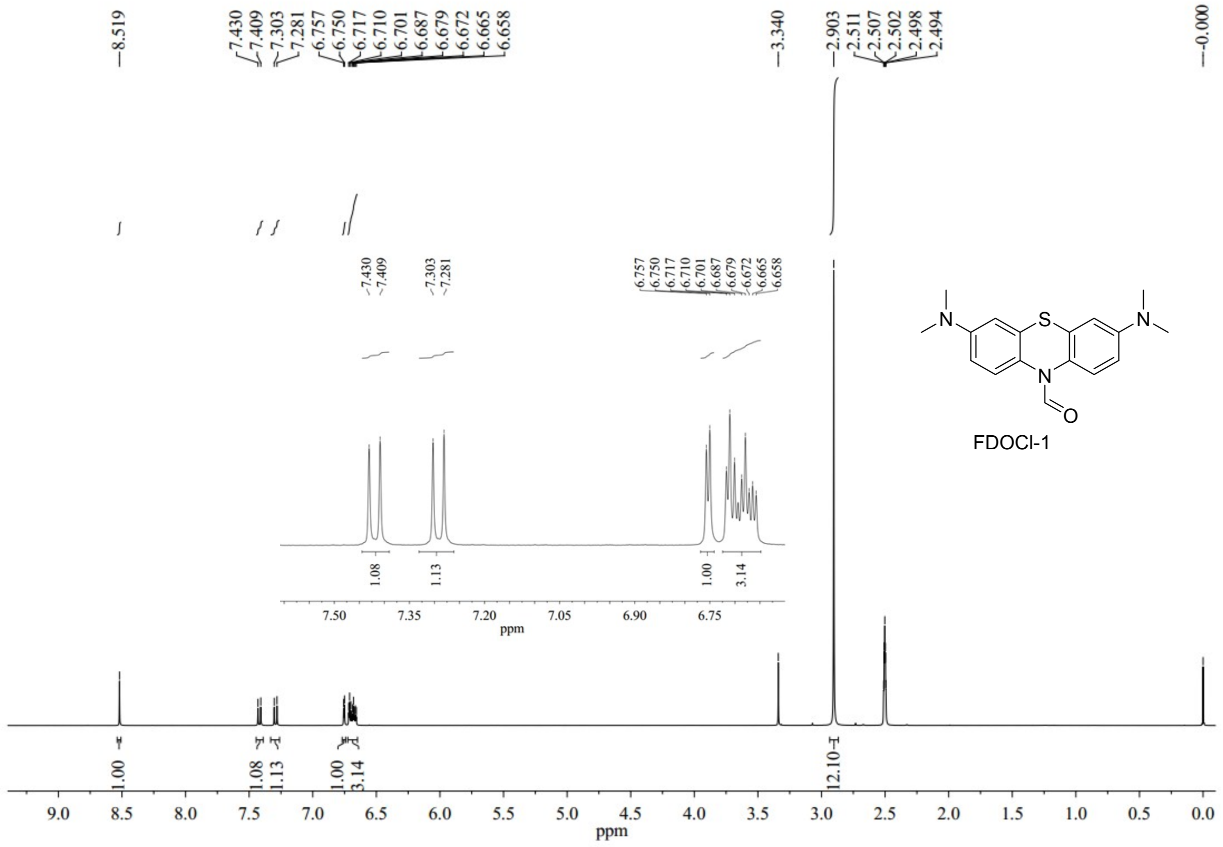

Fig. S25 ^{1}H NMR of **FDOCl-1** in DMSO-d6

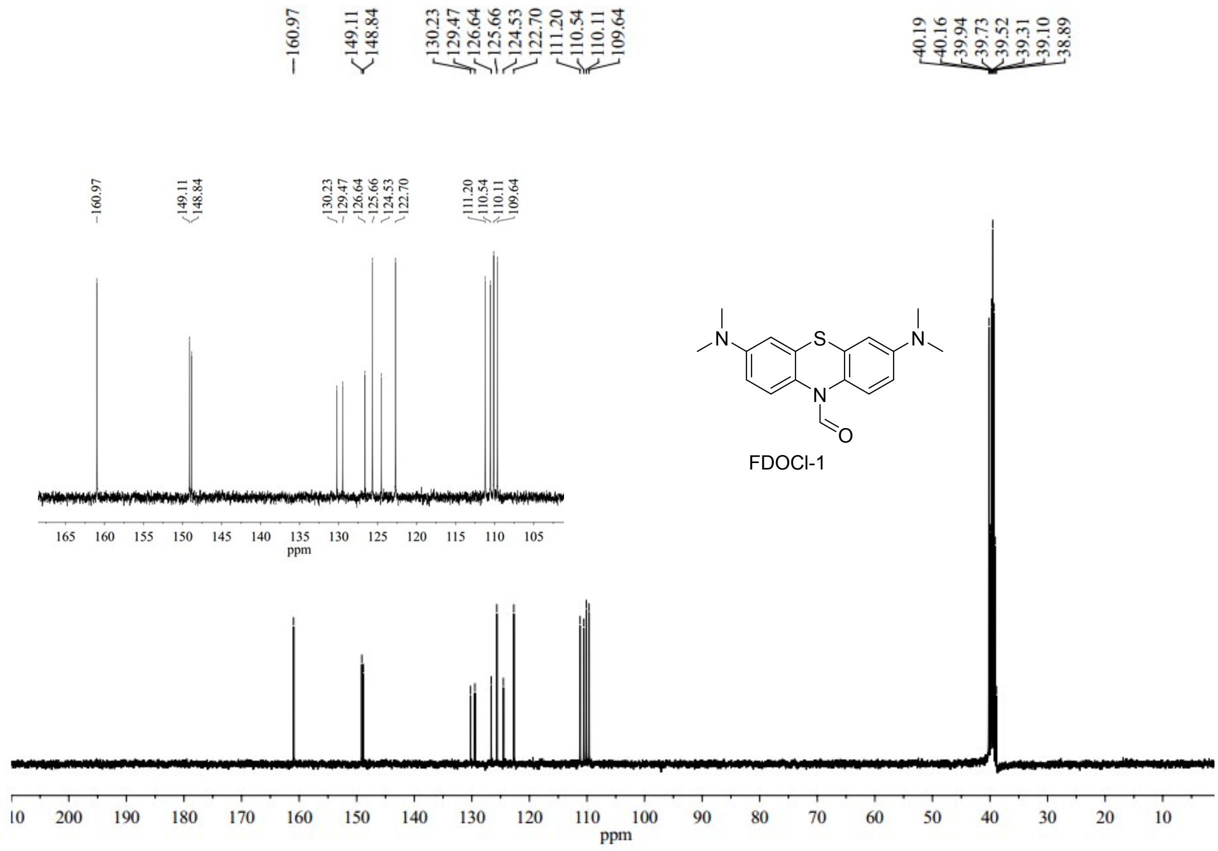

Fig. S26 ^{13}C NMR of **FDOCl-1** in DMSO-d6

Acquisition Parameter					
Source Type	ESI	Ion Polarity	Positive	Set Nebulizer	1.0 Bar
Focus	Not active			Set Dry Heater	200 ℃
Scan Begin	50 m/z	Set Capillary	4000 V	Set Dry Gas	6.0 l/min
Scan End	1000 m/z	Set End Plate Offset	-500 V	Set Divert Valve	Waste

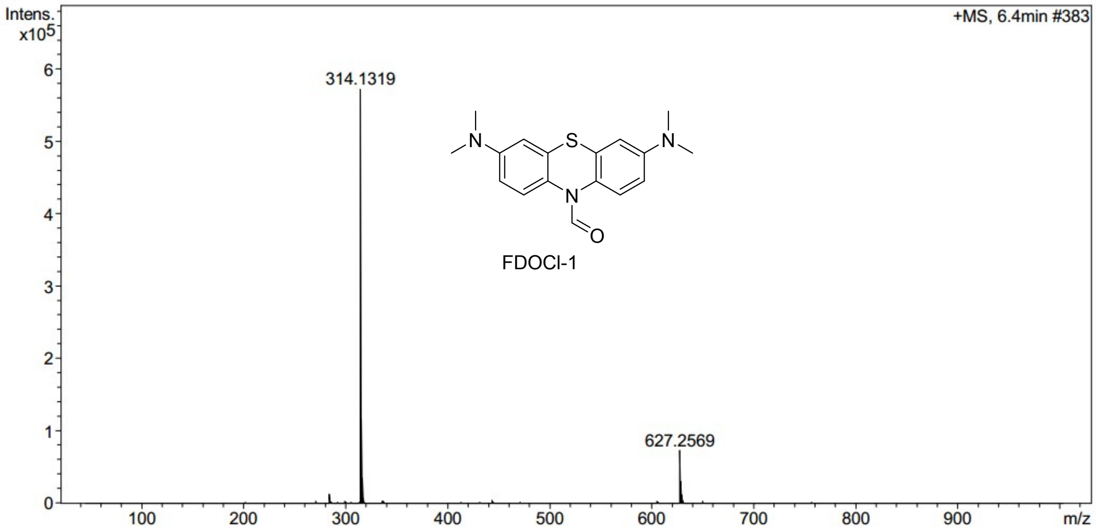

Fig. S27 HRMS of **FDOCl-1**

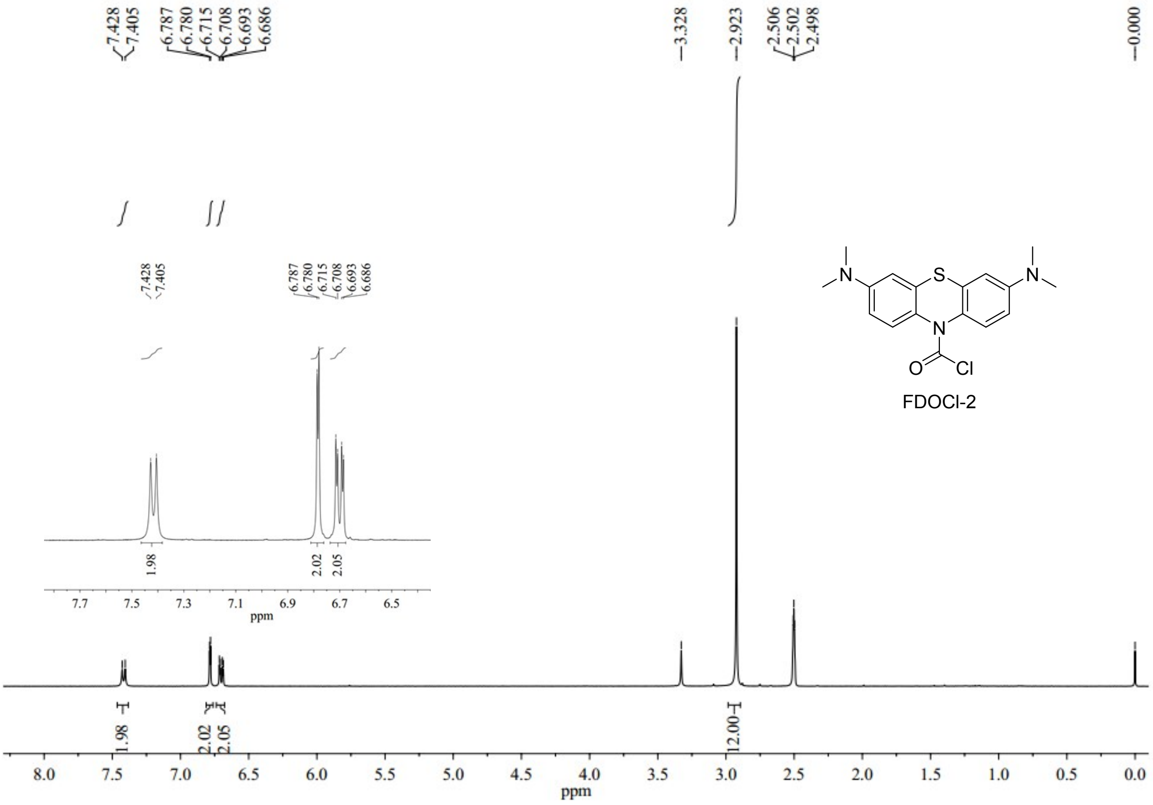

Fig. S28 ^{1}H NMR of **FDOCl-2** in DMSO-d6

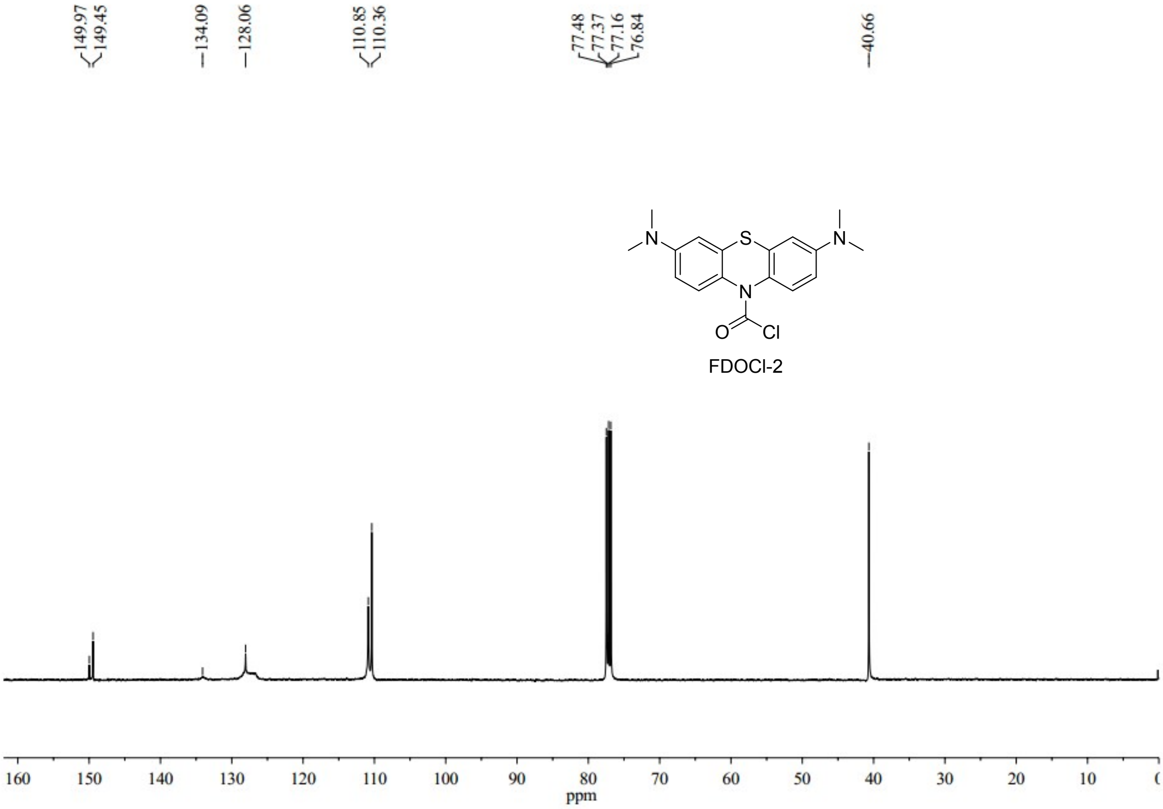

Fig. S29 ^{13}C NMR of **FDOCl-2** in $CDCl_3$

Acquisition Parameter

Source Type	ESI	Ion Polarity	Positive	Set Nebulizer	1.0 Bar
Focus	Not active			Set Dry Heater	200 ℃
Scan Begin	100 m/z	Set Capillary	4000 V	Set Dry Gas	6.0 l/min
Scan End	1500 m/z	Set End Plate Offset	-500 V	Set Divert Valve	Waste

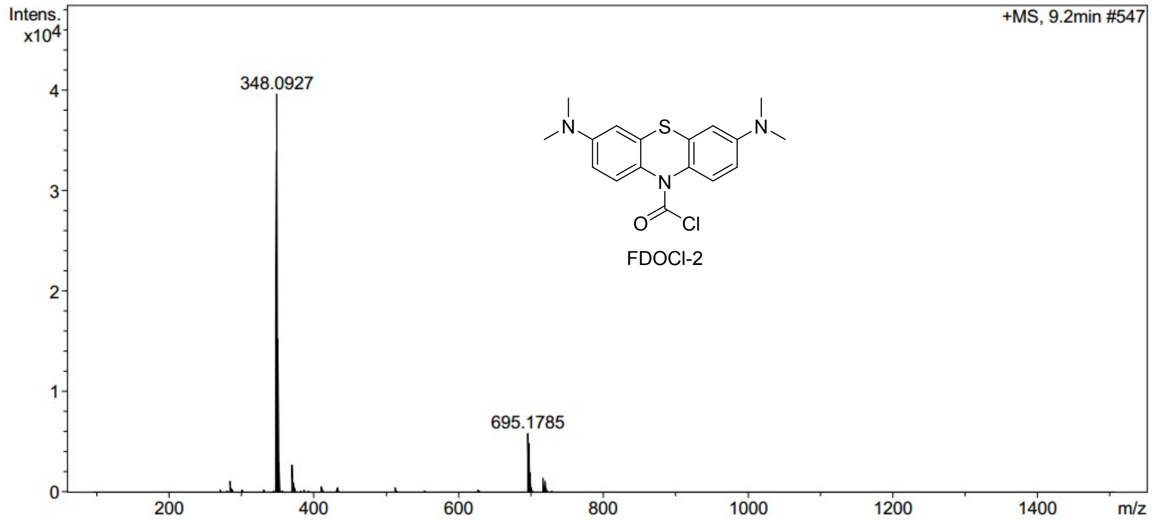

Fig. S30 HRMS of **FDOCl-2**

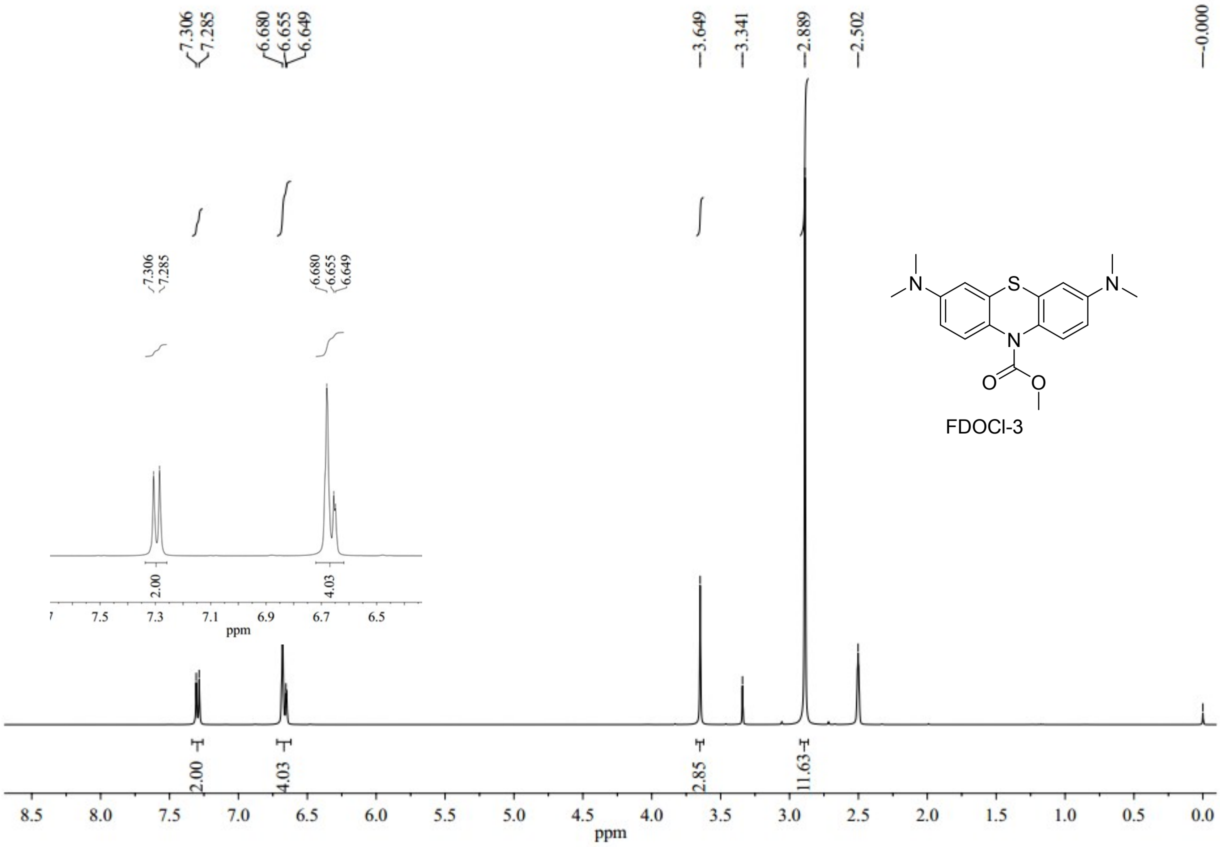

Fig. S31 ^{1}H NMR of **FDOCl-3** in DMSO-d6

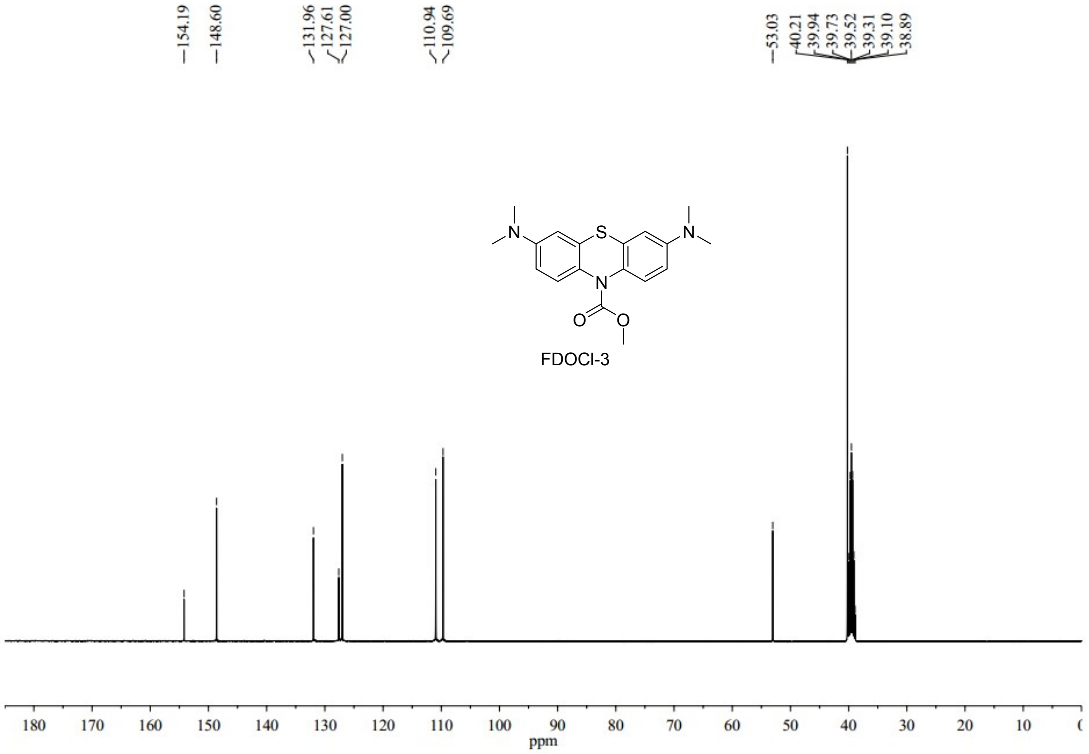

Fig. S32 ^{13}C NMR of **FDOCl-3** in DMSO-d6

Acquisition Parameter

Source Type	ESI	Ion Polarity	Positive	Set Nebulizer	1.0 Bar
Focus	Not active			Set Dry Heater	200 ℃
Scan Begin	100 m/z	Set Capillary	4000 V	Set Dry Gas	6.0 l/min
Scan End	1500 m/z	Set End Plate Offset	-500 V	Set Divert Valve	Waste

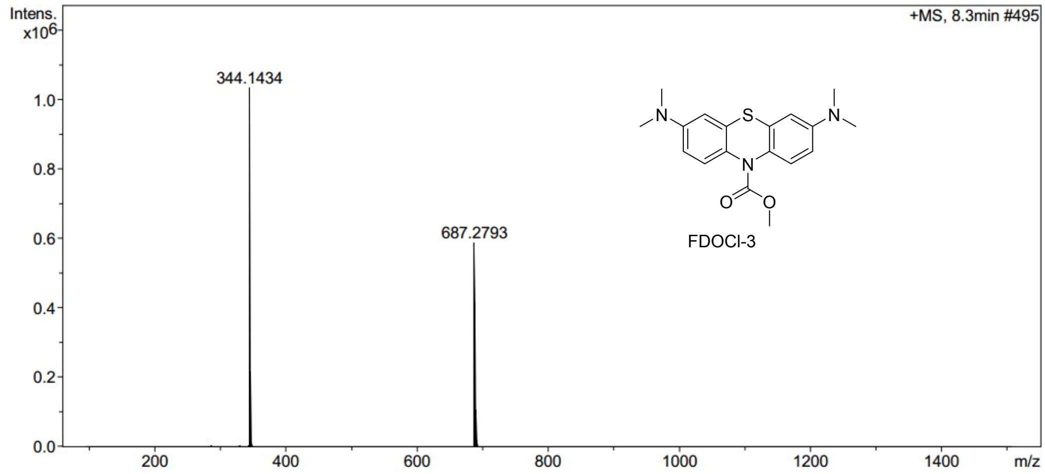

Fig. S33 HRMS of **FDOCl-3**

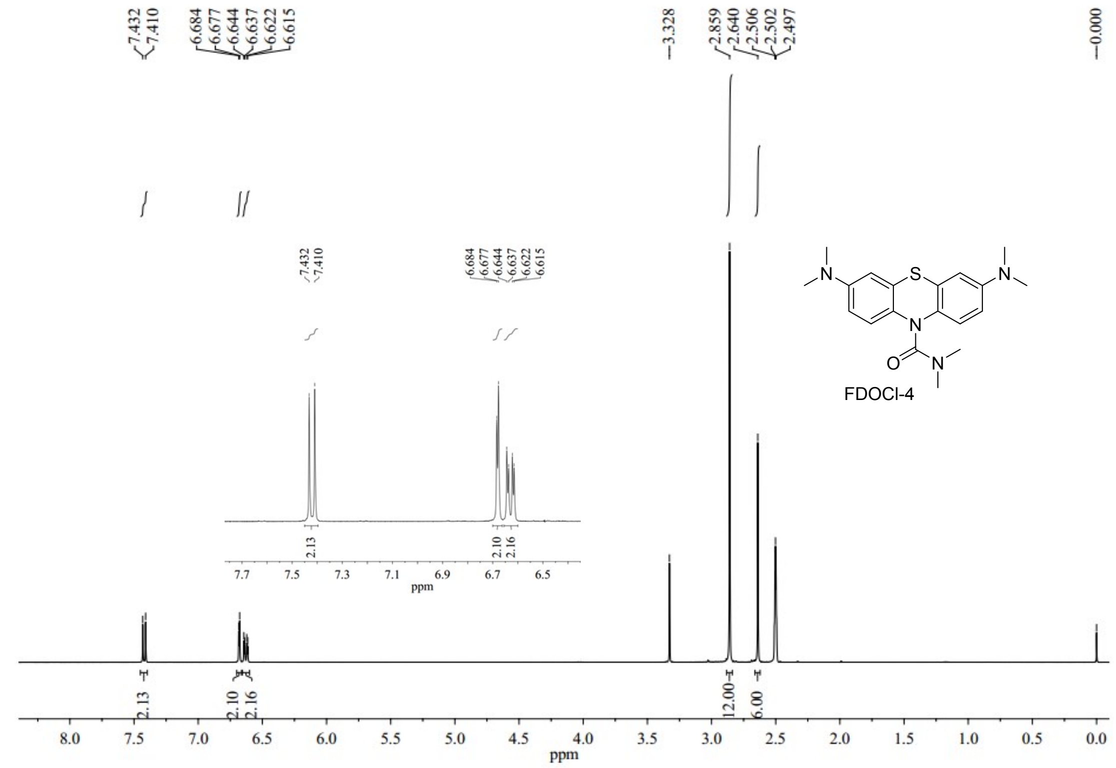

Fig. S34 ^{1}H NMR of **FDOCl-4** in DMSO-d6

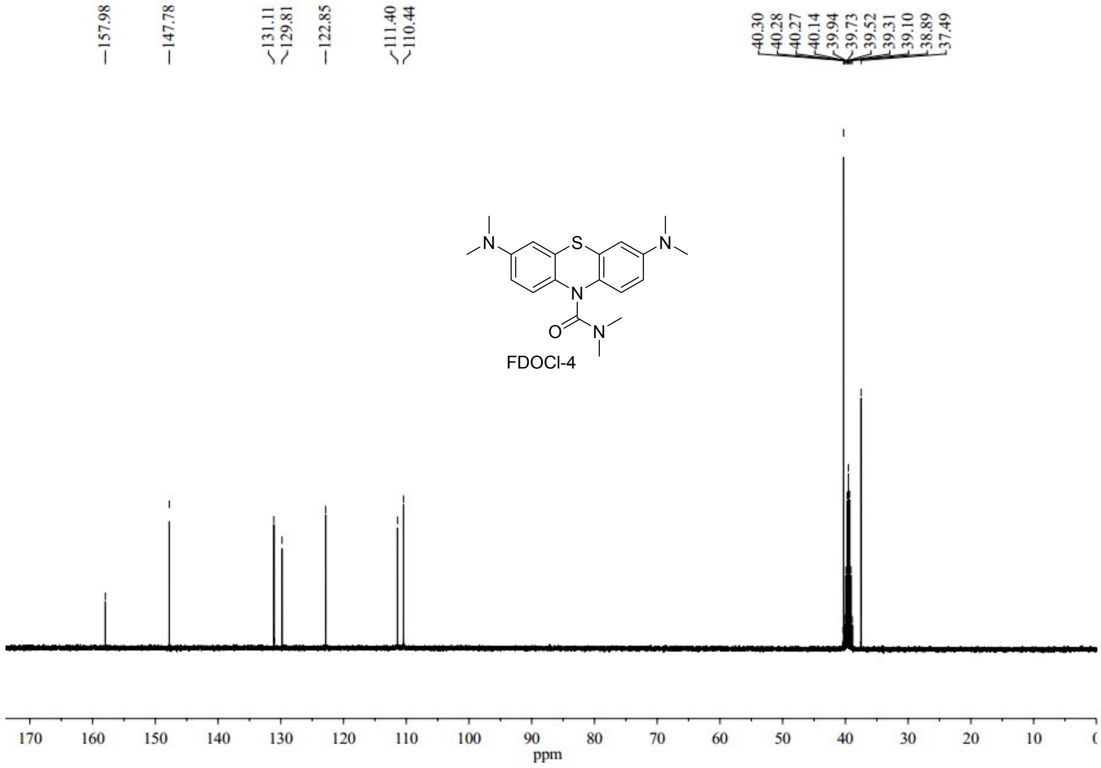

Fig. S35 ^{13}C NMR of **FDOCl-4** in DMSO-d6

Acquisition Parameter

Source Type	ESI	Ion Polarity	Positive	Set Nebulizer	1.0 Bar
Focus	Not active			Set Dry Heater	200 ℃
Scan Begin	100 m/z	Set Capillary	4000 V	Set Dry Gas	6.0 l/min
Scan End	1500 m/z	Set End Plate Offset	-500 V	Set Divert Valve	Waste

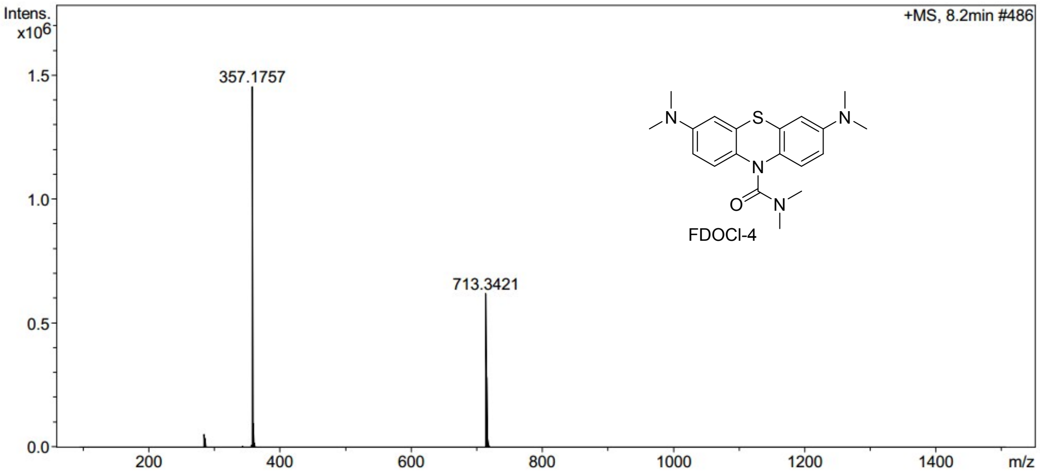

Fig. S36 HRMS of **FDOCl-4**

5 References

1 Z.-N. Sun, F.-Q. Liu, Y. Chen, P. K. H. Tam and D. Yang, *Org. Lett.*, 2008, **10**, 2171-2174.

2 J. J. Hu, N.-K. Wong, Q. Gu, X. Bai, S. Ye and D. Yang, *Org. Lett.*, 2014, **16**, 3544-3547.

3 J. J. Hu, N.-K. Wong, M.-Y. Lu, X. Chen, S. Ye, A. Q. Zhao, P. Gao, R. Yi-Tsun Kao, J. Shen and D. Yang, *Chem. Sci.*, 2016, **7**, 2094-2099.

4 Y. Zhou, J.-Y. Li, K.-H. Chu, K. Liu, C. Yao and J.-Y. Li, *Chem. Commun.*, 2012, **48**, 4677-4679.

5 W. Lin, L. Long, B. Chen and W. Tan, *Chem. – Eur. J.*, 2009, **15**, 2305-2309.

6 X. Cheng, H. Jia, T. Long, J. Feng, J. Qin and Z. Li, *Chem. Commun.*, 2011, **47**, 11978-11980.

7 G. Wu, F. Zeng and S. Wu, *Anal. Methods.*, 2013, **5**, 5589-5596.

8 D. Li, Y. Feng, J. Lin, M. Chen, S. Wang, X. Wang, H. Sheng, Z. Shao, M. Zhu and X. Meng, *Sens. Actuators B: Chem.*, 2016, **222**, 483-491.

9 L. Yuan, W. Lin, Y. Xie, B. Chen and J. Song, *Chem. – Eur. J.*, 2012, **18**, 2700-2706.

10 L. Cao, R. Zhang, W. Zhang, Z. Du, C. Liu, Z. Ye, B. Song and J. Yuan, *Biomaterials*, 2015, **68**, 21-31.

11 X. Chen, K.-A. Lee, E.-M. Ha, K. M. Lee, Y. Y. Seo, H. K. Choi, H. N. Kim, M. J. Kim, C.-S. Cho, S. Y. Lee, W.-J. Lee and J. Yoon, *Chem. Commun.*, 2011, **47**, 4373-4375.

12 X. Chen, K.-A. Lee, X. Ren, J.-C. Ryu, G. Kim, J.-H. Ryu, W.-J. Lee and J. Yoon, *Nat. Protocols*, 2016, **11**, 1219-1228.

13 Q. Xu, K.-A. Lee, S. Lee, K. M. Lee, W.-J. Lee and J. Yoon, *J. Am. Chem. Soc.*, 2013, **135**, 9944-9949.

14 S. Kenmoku, Y. Urano, H. Kojima and T. Nagano, *J. Am. Chem. Soc.*, 2007, **129**, 7313-7318.

15 Y. Koide, Y. Urano, K. Hanaoka, T. Terai and T. Nagano, *J. Am. Chem. Soc.*, 2011, **133**, 5680-5682.

16 L. Yuan, L. Wang, B. K. Agrawalla, S.-J. Park, H. Zhu, B. Sivaraman, J. Peng, Q.-H. Xu and Y.-T. Chang, *J. Am. Chem. Soc.*, 2015, **137**, 5930-5938.

17 G. Li, D. Zhu, Q. Liu, L. Xue and H. Jiang, *Org. Lett.*, 2013, **15**, 2002-2005.

18 Q. Xu, C. H. Heo, G. Kim, H. W. Lee, H. M. Kim and J. Yoon, *Angew. Chem. Int. Ed.*, 2015, **54**, 4890-4894.

19 G. Cheng, J. Fan, W. Sun, J. Cao, C. Hu and X. Peng, *Chem. Commun.*, 2014, **50**, 1018-1020.

20 Z. Lou, P. Li, Q. Pan and K. Han, *Chem. Commun.*, 2013, **49**, 2445-2447.

21 W. Zhang, W. Liu, P. Li, J. kang, J. Wang, H. Wang and B. Tang, *Chem. Commun.*, 2015, **51**, 10150-10153.

22 L. Wu, I. C. Wu, C. C. DuFort, M. A. Carlson, X. Wu, L. Chen, C.-T. Kuo, Y. Qin, J. Yu, S. R. Hingorani and D. T. Chiu, *J. Am. Chem. Soc.*, 2017, **139**, 6911-6918.

23 S. Chen, J. Lu, C. Sun and H. Ma, *Analyst*, 2010, **135**, 577-582.

24 X. Zou, Y. Liu, X. Zhu, M. Chen, L. Yao, W. Feng and F. Li, *Nanoscale*, 2015, **7**, 4105-4113.

25 Y. Zhang, S. Swaminathan, S. Tang, J. Garcia-Amorós, M. Boulina, B. Captain, J. D. Baker and F. M. Raymo, *J. Am. Chem. Soc.*, 2015, **137**, 4709-4719.
